# New Nanostructured Materials Based on Mesoporous Silica Loaded with Ru(II)/Ru(III) Complexes with Anticancer and Antimicrobial Properties

**DOI:** 10.3390/pharmaceutics15051458

**Published:** 2023-05-10

**Authors:** Gabriela Marinescu, Daniela C. Culita, Teodora Mocanu, Raul-Augustin Mitran, Simona Petrescu, Miruna S. Stan, Mariana C. Chifiriuc, Marcela Popa

**Affiliations:** 1 Ilie Murgulescu Institute of Physical Chemistry, 202 Splaiul Independentei, 060021 Bucharest, Romania; gmarinescu@icf.ro (G.M.); tmocanu@icf.ro (T.M.); mitran@icf.ro (R.-A.M.); simon_pet@yahoo.com (S.P.); 2 Faculty of Biology, University of Bucharest, 90 Panduri Street, 050663 Bucharest, Romania; miruna.stan@bio.unibuc.ro (M.S.S.); carmen.chifiriuc@bio.unibuc.ro (M.C.C.); marcela.popa@bio.unibuc.ro (M.P.)

**Keywords:** ruthenium complexes, SBA-15, Schiff bases, nanostructured hybrid materials, lung cancer cells, antitumor activity, antibiofilm, antibacterial

## Abstract

A new series of nanostructured materials was obtained by functionalization of SBA-15 mesoporous silica with Ru(II) and Ru(III) complexes bearing Schiff base ligands derived from salicylaldehyde and various amines (1,2-diaminocyclohexane, 1,2-phenylenediamine, ethylenediamine, 1,3-diamino-2-propanol, N,N-dimethylethylenediamine, 2-aminomethyl-pyridine, and 2-(2-aminoethyl)-pyridine). The incorporation of ruthenium complexes into the porous structure of SBA-15 and the structural, morphological, and textural features of the resulting nanostructured materials were investigated by FTIR, XPS, TG/DTA, zeta potential, SEM, and N_2_ physisorption. The ruthenium complex-loaded SBA-15 silica samples were tested against A549 lung tumor cells and MRC-5 normal lung fibroblasts. A dose-dependent effect was observed, with the highest antitumoral efficiency being recorded for the material containing [Ru(Salen)(PPh_3_)Cl] (50%/90% decrease in the A549 cells’ viability at a concentration of 70 μg/mL/200 μg/mL after 24 h incubation). The other hybrid materials have also shown good cytotoxicity against cancer cells, depending on the ligand included in the ruthenium complex. The antibacterial assay revealed an inhibitory effect for all samples, the most active being those containing [Ru(Salen)(PPh_3_)Cl], [Ru(Saldiam)(PPh_3_)Cl], and [Ru(Salaepy)(PPh_3_)Cl], especially against *Staphylococcus aureus* and *Enterococcus faecalis* Gram-positive strains. In conclusion, these nanostructured hybrid materials could represent valuable tools for the development of multi-pharmacologically active compounds with antiproliferative, antibacterial, and antibiofilm activity.

## 1. Introduction

One of the greatest challenges standing in front of modern biomedical science is the resistance to both antibacterial and antitumoral agents, raising an acute necessity to develop new, safe, and highly effective therapeutic strategies. Nowadays, the treatment of any malignancy is based on surgery, radiotherapy, and chemotherapy. Despite significant progress in understanding the molecular biology of cancer development, the design of novel cytotoxic anticancer drugs continues to be the cornerstone of modern antitumor therapy. After the discovery of cisplatin in 1960, the use of metallodrugs to treat cancer has been a great development. Since then, many metal-based drugs have been investigated for their activity against various types of cancer. Despite the discovery of antibiotics and vaccines, infectious diseases are still one of the top causes of mortality and morbidity, challenging the success of many tools in modern medicine such as surgery, tissue engineering, or oncological treatments through the emergence and spread of resistance to all currently used antibiotics and the development of microbial biofilms on living tissues and medical devices. Metal complexes offer promising leads for the development of antimicrobial and anti-biofilm disrupting agents due to their multiple molecular targets and mechanisms of action. However, in many cases, it was not possible to exploit all the characteristics of the metal complexes because some of the most promising ones proved to have severe adverse effects and, in most cases, had low stability in aqueous solutions and, thus, low bioavailability [1]. Hence, the problems associated with the use of metal-based drugs for cancer and infectious diseases treatment have stimulated the search for new alternatives based on different metals and ligands with enhanced bioactivity.

Recent research studies have highlighted a number of ruthenium-based compounds with very promising anticancer properties that can serve as viable alternatives to cisplatin and its derivatives [2,3,4,5]. For example, three Ru(III) complexes, NAMI-A, KP1019, and NKP-1339, have entered clinical trials for cancer treatment [6,7,8]. Ruthenium complexes have unique and versatile biochemical properties, and many of them have low general toxicity toward healthy tissues [9,10]. One of the mechanisms of action of ruthenium complexes consists of their interaction with DNA, explaining both the anticancer but also the antimicrobial properties, which have been recently highlighted, suggesting their potential as antibacterial, antifungal, antiparasitic, or antiviral drugs [11,12]. However, some of the ruthenium complexes present drawbacks, mainly because they can be deactivated by binding to proteins present in the blood or by hydrolysis. In some cases, their limited solubility in water can make difficult their intravenous administration and may also lead to a weak therapeutic effect. Many ruthenium complexes have a limited capacity to cross the cell membrane. For these reasons, alternative methods to deliver these types of drugs are essential for maximizing the therapeutic performance of newly developed metal-based drugs.

Nanocarriers, which play a significant role in achieving the desired effectiveness, can modulate the way a substance enters the body, accumulates in different anatomic regions, and interacts with target tissues and cells. The delivery process can be adapted to the specific drug by adjusting the properties of the nanocarrier. This aspect is especially important when the compounds are cytotoxic or poorly soluble in water, which causes reduced activity or severe adverse effects before reaching the intended target [13]. Different nanostructured systems, each with its advantages and drawbacks, have been investigated in recent years for their capability to function as delivery systems for metal-based drugs: liposomes, lipid nanocapsules, cucurbit[n]urils cyclodextrins, mesoporous silica nanoparticles, carbon nanotubes, polymeric nanoparticles, etc. [14]. Because of its remarkable and unique features, such as large specific surface area and pore volume, tunable pore sizes, ease of functionalizing, good biocompatibility, and lack of toxicity, mesoporous silica shows great promise as a feasible platform for delivering hydrophobic drugs, acting as a universal transmembrane carrier for intracellular drug delivery and imaging applications [15]. Nanostructured mesoporous silica materials such as MCM-41 and SBA–15, loaded with different biologically active compounds, have been extensively studied and proposed for a wide range of biological applications. A variety of drugs such as anti-inflammatory, bactericidal, anti-hypertensive, anti-depressant, anti-ulcer, and anti-osteoporotic have been loaded onto these materials for delivery purposes [16,17,18]. The first study that involved the functionalization of mesoporous silica with anticancer metallodrugs was carried out with cisplatin [19]. Other research groups have reported titanocene-functionalized MCM-41 or SBA–15 with very promising antitumor activity [20]. As far as we know, there are only a few publications on the utilization of ruthenium compounds loaded onto mesoporous silica as cytotoxic agents against cancer and bacterial cells [14,21,22,23,24,25,26,27,28,29]. A novel cancer-targeted nanodrug delivery system based on RGD peptide-conjugated MSNs loaded with a fluorescent ruthenium complex ([Ru(phen)_2_-p-MOPIP](PF_6_)_2_·2H_2_O) has been reported by He et al. [30]. This system allows the direct fluorescence monitoring of the cellular uptake and releases of ruthenium complex in cancer cells and dramatically enhances the anticancer efficacy of the hydrophobic ruthenium complex [30]. Sun et al. fabricated a ruthenium-loaded palmitoyl ascorbate (PA)-modified mesoporous silica that showed promising activity against human cancer cells in vitro and in vivo [31]. Martinez-Carmona et al. reported that the material obtained by encapsulation of [Ru(ppy-CHO)(phen)_2_][PF_6_] in mesoporous silica nanoparticles functionalized with amino groups shows very high anticancer activity against U87 glioblastoma cells [13]. Harun et al. demonstrated that encapsulation of novel ruthenium polypyridyl complexes (Ru-PIP) in mesoporous silica enhances significantly the cytotoxicity against Hela, A549, and T24 cancer cell lines, compared to unloaded Ru-PIP [32].

In this context and in continuation of our research work in the field of materials with biological activity, the aim of our study was to develop a new series of hybrid nanosystems based on Ru(II)/Ru(III) complexes with Schiff base ligands loaded in mesoporous silica and to evaluate their antimicrobial and anticancer properties. We were encouraged by the results obtained in one of our previous studies, in which the hybrid materials constructed through the immobilization of three Ru(III) complexes bearing Schiff base ligands derived from o-vanillin inside the mesoporous channels of SBA-15 exhibited very good cytotoxic activity against HeLa tumor cells [33].

## 2. Materials and Methods

### 2.1. Materials

Tetraethoxysilane > 99% (TEOS), 3-aminopropyl-trimethoxysilane > 99% (APTES), ruthenium(III) chloride hydrate, triphenyl phosphine > 97%, o-vanillin > 98%, salicylaldehyde > 99%, ethylenediamine > 99%, hydrochloric acid 2M, methanol > 99.9% p.a., dichloromethane > 99.8% (CH_2_Cl_2_) (Merck Millipore, Darmstadt, Germany), Triblock copolymer Pluronic P123 (Poly(ethylene glycol)-block-poly(propylene glycol)-block-poly(ethylene glycol)) average Mn ~5800, 1,2-diaminocyclohexane 99%, 2-(2-aminoethyl)pyridine 99%, 2-(aminomethyl)pyridine > 99%, N,N-dimethylethylenediamine 98%, 1,2-phenylenediamine > 99%, 1,3-diamino-2-propanol > 95% (Sigma-Aldrich, Darmstadt, Germany), and dry toluene ≥ 99.5% (≤50 ppm H_2_O) (Carl Roth, Karlsruhe, Germany) were used as received.

### 2.2. Characterization Methods

FT-IR spectra on KBr pellets were acquired using a Jasco FT/IR-4700 spectrophotometer (Tokyo, Japan). UV-Vis spectra were recorded using a JASCO V-750 spectrophotometer (Tokyo, Japan). Thermogravimetric analyses (TGA) coupled with differential thermal analyses (DTA) were performed using a Mettler Toledo TGA/SDTA851e thermogravimeter (Greifensee, Switzerland), under 80 mL min^−1^ synthetic air atmosphere, at a heating rate of 10 °C min^−1^. Sample composition was computed from the mass loss curves, with respect to the dry sample mass at 110 °C. A Micromeritics ASAP 2020 analyzer (Norcross, GA, USA) was used to measure the N_2_ adsorption–desorption isotherms at −196 °C. Before analysis, the samples were heated at 80 °C for 6 h under vacuum to remove all of the adsorbed species. Specific surface areas (S_BET_) were calculated using the Brunauer–Emmett–Teller (BET) method, while the amount adsorbed at a relative pressure of 0.99 was used to compute the total pore volume (V_total_). The Barrett–Joyner–Halenda (BJH) method was applied to obtain the average pore diameter using the desorption data. Elemental analysis (C, H, N) was performed using an EuroEA elemental analyzer (HEKAtech GmbH, Wegberg, Germany). The magnetic properties were assessed at room temperature on a fully integrated Vibrating Sample Magnetometer system 7404 from Lake Shore (Westerville, OH, USA). XPS analysis was performed on a Kratos Ultra DLD Setup (Kratos Analytical Ltd., Manchester, UK) using a monochromatic Al-Kα source (hν = 1486.74 eV, X-ray source). A charge neutralizer was used for all samples and the conditions for recording XP spectra were as follows: power 240 W (20 kV × 12 mA), pressure 1 × 10^−7^ Pa. The samples were calibrated to 284.6 eV (C 1s). Zeta potential measurements were performed on a Backman Coulter Delsa Nano C analyzer (Brea, CA, USA), at 25 °C. All samples for zeta potential measurements were suspended in water at a concentration of 250 μg mL^−1^. The morphology of the samples was analyzed by scanning electron microscopy (SEM) using a FEI Quanta 3D FEG microscope (FEI, Brno, Czech Republic).

### 2.3. Synthesis of the Materials

**Figure 1:**
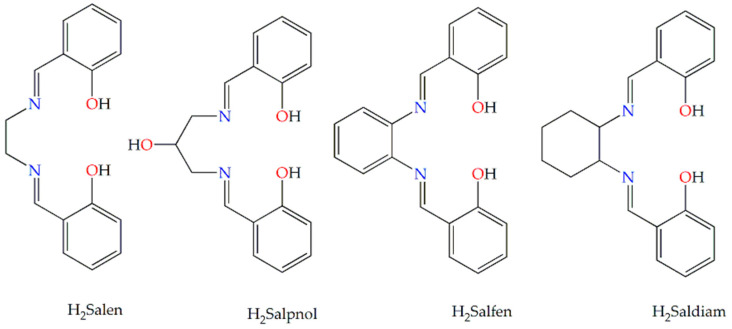
Structure of compartmental Schiff base proligands H_2_L^n^.

**Figure 2:**
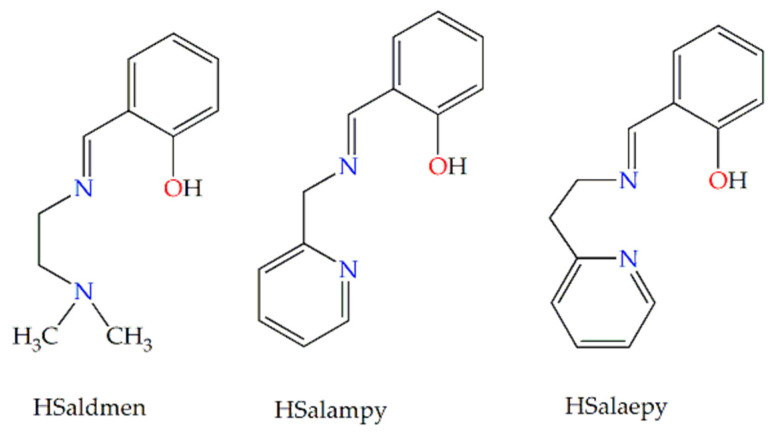
Structure of Schiff base proligands HL^m^.

The compartmental Schiff base proligands (H_2_L^n^) (Figure 1) were synthesized by the condensation of salicylaldehyde with ethylenediamine (H_2_Salen), 1,3-diamino-2-propanol (H_2_Salpnol), 1,2-phenylenediamine (H_2_Salfen), and 1,2-diaminocyclohexane (H_2_Saldiam), respectively, in ethanol. In the case of organic proligands HL^m^ (Figure 2), the synthetic procedure consisted of in situ formation of Schiff bases by condensation reactions of salicylaldehyde with organic molecules bearing only one primary amino group, N,N-dimethylethylenediamine (HSaldmen), 2-aminomethyl-pyridine (HSalampy), and 2-(2-aminoethyl)-pyridine (HSalaepy), respectively, in ethanol, as in our previous research articles [34,35,36]. The starting complexes, [Ru^II^(PPh_3_)_3_Cl_2_] and [Ru^III^(Salen)(PPh_3_)Cl], were synthesized according to the literature methods with minor modifications [37,38].

#### 2.3.1. Synthesis of Ru(III) Complexes

All the Ru(III) complexes, [Ru^III^(L^n^)(PPh_3_)Cl]·xH_2_O, were synthesized following Murray’s synthesis procedure [38] with minor modifications, using H_2_Salpnol, H_2_Salphen, and H_2_Saldiam instead of H_2_Salen.

Synthesis of [Ru(Salen)(PPh_3_)Cl] (RuSalen): The mononuclear Ru(III) complex was synthesized by the reaction between [Ru(PPh_3_)_3_Cl_2_] (1 mmol) and H_2_Salen (1 mmol) in methanol, at 60 °C. An excess of triethylamine (Et_3_N) was added with continuous stirring to the resulting green solution, and the reaction was carried out in the presence of air to ensure the oxidation of Ru^II^ ions. A green–black precipitate was extracted after the addition of diisopropyl ether. The solid product was collected by filtration, washed several times with water and toluene to remove Et_3_N⸳HCl and PPh_3_, then dried in air. Single crystals suitable for X-ray diffraction were obtained by dissolving the green–black powder into a mixture dichloromethane/diethyl ether (1:1). Slow evaporation of the reaction mixture gave a crystalline product after a few days. IR data (KBr, cm^−1^): 3058 m, 3008 m, 2954 m, 2923 m, 2738 m, 1603 vs, 1528 s, 1481 m, 1434 vs, 1340 m, 1295 s, 1194 m, 1146 m, 1130 w, 1092 s, 1029 m, 997 m, 902 w, 851 w, 787 w, 749 s, 696 vs, 598 w, 522 vs, 461 w. Elemental chemical analysis (%) for C_34_H_29_N_2_O_2_ClPRu: C, 61.34; H, 4.36; N, 4.21 (calcd); C, 60.91; H, 5.05; N, 4.91 (found).

Compounds [Ru(Salpnol)(PPh_3_)Cl]·H_2_O (RuSalpnol), [Ru(Salfen)(PPh_3_)Cl] (RuSalfen), [Ru(Saldiam)(PPh_3_)Cl]·H_2_O (RuSaldiam), were obtained following the same general procedure described for RuSalen, using H_2_Salpnol, H_2_Salfen, and H_2_Saldiam instead of H_2_Salen. These compounds were obtained as dark-green precipitates. Since single crystals suitable for X-ray diffraction could not be obtained for these compounds, their structures were proved by elemental analyses, FTIR and UV-Vis spectroscopy, and magnetic measurements at room temperature.

RuSalpnol: IR data (KBr, cm^−1^): 3400 m, 3053 m, 2976 s, 2936 s, 2738 m, 2677 s, 2603 sh, 2492 s, 1600 vs, 1521 s, 1480 s, 1434 vs, 1398 s, 1306 m, 1187 w, 1149 w, 1093 s, 1036 s, 902 w, 849 w, 805 w, 752 s, 696 vs, 615 w, 524 vs, 460 w. Elemental chemical analysis (%) for C_35_H_33_N_2_O_3_ClPRu: C, 60.29; H, 4.73; N, 4.01 (calcd); C, 59.19; H, 3.98; N, 4.96 (found).

RuSalfen: IR data (KBr, cm^−1^): 3054 m, 2976 m, 2939 m, 2738 m, 2675 m, 2603 m, 2498 m, 1600 vs, 1567 s, 1518 vs, 1481 s, 1456 s, 1432 vs, 1397 vw, 1314 s, 1182 s, 1146 s, 1127 vw, 1092 s, 1035 m, 997 w, 924 m, 850 s, 799 vs, 744 vs, 695 vs, 616 w, 558 w, 539 s, 524 vs, 463 w. Elemental chemical analysis (%) for C_38_H_29_N_2_O_2_ClPRu: C, 63.99; H, 4.06; N, 3.92 (calcd); C, 62.88; H, 3.79; N, 4.28 (found).

RuSaldiam: IR data (KBr, cm^−1^): 3417 m, 3055 m, 2980 m, 2933 m, 2742 w, 2676 m, 2603 sh, 2496 m, 1597 vs, 1527 s, 1481 m, 1461 w, 1433 vs, 1397 w, 1318 m, 1302 s, 1188 s, 1148 m, 1093 m, 1034 w, 997 w, 899 m, 853 m, 806 w, 753 s, 695 vs, 608 w, 525 m, 458 w. Elemental chemical analysis (%) for C_38_H_37_N_2_O_3_ClPRu: C, 61.90; H, 5.02; N, 3.80 (calcd); C, 60.85; H, 4.90; N, 4.29 (found).

#### 2.3.2. Synthesis of Ru(II) Complexes

All of the Ru(II) complexes, [Ru^II^(L^m^)(PPh_3_)Cl], were synthesized following the same general synthetic procedure, with a mention that in this case the Schiff base proligands were synthesized in situ.

Synthesis of [Ru(Saldmen)(PPh_3_)Cl] (RuSaldmen): Ethanolic solutions containing stoichiometric amounts of salicylaldehyde (1 mmol, 10 mL) and N,N-dimethyl-ethylene diamine (1 mmol, 5 mL) were mixed and kept under continuous stirring for 4 h at 60 °C. The resulting yellow solution was treated with an excess of triethylamine (in 5 mL EtOH) and then 1 mmol [Ru(PPh_3_)_3_Cl_2_] (solid) was added. The mixture was refluxed at 60 °C for 4 h under continuous stirring. A brown–green diamagnetic precipitate was extracted after addition of diisopropyl ether. The solid product was collected by filtration, washed several times with water and toluene to remove Et_3_N⸳HCl and PPh_3_, and dried in the air. IR data (KBr, cm^−1^): 3391 m, 3050 m, 2980 m, 2923 m, 2738 m, 2669 vw, 1598 vs, 1536 s, 1467 m, 1434 vs, 1398 sh, 1338 w, 1288 m, 1189 m, 1149 m, 1128 w, 1090 m, 1027 w, 997 w, 943 w, 910 m, 891 w, 797 vw, 750 s, 695 vs, 625 w, 593 w, 517 vs, 452 w. Elemental chemical analysis (%) for C_29_H_30_N_2_OClPRu: C, 59.03; H, 5.08; N, 4.74 (calcd); C, 61.02; H, 4.99; N, 4.02 (found).

Compounds [Ru(Salampy)(PPh_3_)Cl] (RuSalampy), and [Ru(Salaepy)(PPh_3_)Cl] (RuSalaepy) were obtained following the same synthetic protocol described for RuSaldmen complex, using 2-aminomethyl-pyridine (HSalampy), and 2-(2-aminoethyl)-pyridine (HSalaepy), instead of N,N-dimethyl-ethylenediamine (HSaldmen). These compounds were obtained as brown-green diamagnetic precipitates. Single crystals suitable for X-ray diffraction could not be obtained for these compounds. Their structures were proven by elemental analyses, FTIR and UV-Vis spectroscopy, and magnetic measurements at room temperature.

RuSalampy: IR data (KBr, cm^−1^): 3392 m, 3199 w, 3050 m, 2977 w, 2944 w, 2743 vw, 2669 m, 2496 m, 2359 m, 1597 s, 1541 m, 1505 m, 1480 s, 1433 vs, 1397 sh, 1342 m, 1278 m, 1187 m, 1156 w, 1124 w, 1091 m, 1058 w, 1039 m, 1010 w, 850 w, 748 s, 695 vs, 618 w, 530 s, 515 vs, 458 w. Elemental chemical analysis (%) for C_31_H_26_N_2_OClPRu: C, 61.03; H, 4.26; N, 4.59 (calcd); C, 60.73; H, 5.01; N, 4.12 (found).

RuSalaepy: IR data (KBr, cm^−1^): 3399 m, 3052 m, 2980 w, 2941 w, 2676 w, 2484 w, 2350 w, 1595 vs, 1536 s, 1479 vs, 1434 vs, 1287 s, 1185 w, 1154 w, 1090 m, 1028 w, 997 w, 901 w, 750 m, 696 vs, 529 vs, 514 s, 455 w. Elemental chemical analysis(%) for C_32_H_28_N_2_OClPRu: C, 61.70; H, 4.42; N, 4.42 (calcd); C, 61.22; H, 4.09; N, 3.98 (found).

#### 2.3.3. Synthesis of SBA–15 and SBA15–NH_2_

The SBA–15 synthesis method was adopted from the reports already published, via a sol-gel hydrothermal process, using Pluronic P123 as a surfactant (template) and TEOS as a silica precursor, in an acidic solution, filtered, dried, and then thermally threated at 550 °C for 6 h with a heating rate of 1 °C min^−1^ to eliminate the template [39]. To obtain SBA15–NH_2_, 0.65 ml APTES was added drop-by-drop to a dispersion of 1.2 g SBA–15 in 45 ml dry toluene, at 110 °C for 20 h, then the white solid formed was separated by centrifugation, washed several times with methylene chloride (CH_2_Cl_2_), and dried at 60 °C.

#### 2.3.4. Immobilization of Ruthenium Complexes on SBA–15

SBA15–NH_2_ (0.15 g) was added to a solution of [Ru^III^(L^n^)(PPh_3_)Cl]/[Ru^II^(L^m^)(PPh_3_)Cl] complexes (0.05 g) dissolved in CH_2_Cl_2_ (30 mL), and the mixture was stirred at 40 °C for 2 days to give a green/brown–green solid that was separated from the suspension, washed three times with CH_2_Cl_2_ to remove the excess of ruthenium complex, and then dried in air. The obtained materials were designated as follows: SBA15–RuSalpnol, SBA15–RuSalen, SBA15–RuSaldiam, SBA15–RuSalfen, SBA15–RuSaldmen, SBA15–RuSalampy, and SBA15–RuSalaepy.

### 2.4. Biological Evaluation

#### 2.4.1. Antibacterial Activity Assay

The antibacterial activity of the functionalized mesoporous silica was evaluated against four standard strains: *Staphylococcus aureus* ATCC 25923, *Enterococcus faecalis* ATCC 29212, *Escherichia coli* ATCC 25922, and *Pseudomonas aeruginosa* ATCC 27853.

The qualitative evaluation of the antimicrobial activity was performed following the CLSI (Clinical and Laboratory Standards Institute, Berwyn, PA, USA) guidelines using the agar diffusion method. Briefly, inoculums with a turbidity adjusted to 0.5 McFarland were prepared from fresh cultures and inoculated on Mueller–Hinton agar plates. A volume of 10 μL of each compound was placed on the agar surface, and after overnight incubation at 37 °C the growth inhibition zones diameters were measured with a ruler.

The quantitative analysis of the antimicrobial activity was carried out using the broth microdilutions assay. Two-fold dilutions of the Ru(II)- and Ru(III)-based compounds were prepared in culture liquid medium distributed in a 96-well plate, with the tested concentrations ranging from 5 to 0.002 mg/mL. Ciprofloxacin was used as a positive control. Each well was inoculated with a bacterial inoculum of 10^6^ CFU/mL (colony forming units). Sterility controls and growth controls were used in order to determine the inhibitory effect. After overnight incubation at 37 °C, the bacterial growth was evaluated by reading the optical density at 620 nm (Multiskan FC Thermo Scientific, Waltham, MA, USA). The minimum inhibitory concentration (MIC) was determined as the lowest concentration that inhibits bacterial growth. The assays were performed in duplicate and the results were presented as mean ± standard deviation (SD).

In order to determine the compounds’ interference with the bacterial adherence to inert substrata and the subsequent biofilm development, the crystal violet assay was used. After MIC determination, the 96-well plates were discarded, washed with phosphate buffered saline, and fixed with cold methanol for 5 min in order to fix the adhered bacterial cells, which were further stained with 1% crystal violet solution for 20 min. Following the removal of the dye, a 33% acetic acid solution was added in each well, and after 10 min, the absorbance at 492 nm was read using a plate-reading spectrophotometer (Multiskan FC Thermo Scientific, Waltham, MA, USA). The assays were performed in duplicate and the results were presented as mean ± standard deviation (SD).

#### 2.4.2. Cytotoxicity Assay

Human lung cancer cells (A549 cell line) and human non-tumoral lung fibroblasts (MRC-5 cell line) were purchased from the American Type Culture Collection (ATCC, Manassas, VA, USA) and grown in Dulbecco Modified Eagle’s Medium and Eagle’s Minimum Essential Medium, respectively (Gibco, Thermo Fischer Scientific, Waltham, MA, USA) with 10% fetal bovine serum (Gibco, Thermo Fischer Scientific, Waltham, MA, USA) at 37 °C in a humidified atmosphere with 5% CO_2_. The cells were seeded in 96-well plates at a cell density of 2 × 10^4^ cells/well and left to adhere overnight. The SBA–15- and Ru-based suspensions prepared in cell culture medium were incubated with the attached cells at different concentrations (0, 10, 35, 70, 100, and 200 μg/mL) for 24 and 72 h. After each period of exposure, the cell viability and the nitric oxide (NO) level were measured and compared to control.

The viability was quantified after incubating the cells with 1 mg/mL of 3-(4,5-dimethylthiazol-2-yl)-2,5-diphenyltetrazolium bromide (MTT, Sigma-Aldrich, Burlington, MA, USA) solution for 2 h at 37 °C. The purple formazan crystals formed in the live cells were dissolved with 2-propanol (Sigma-Aldrich, Burlington, MA, USA) and the absorbance was measured at 595 nm using a plate multireader (FlexStation 3, Molecular Device, San Jose, CA, USA). Compound concentrations that produce 50% cell growth inhibition (IC_50_) were calculated from curves constructed by plotting cell survival (%) versus drug concentration (μg/mL) using the Quest Graph™ IC50 calculator (AAT Bioquest, Pleasanton, CA, USA).

The level of nitric oxide (NO) released in the culture medium was quantified with the Griess reagent, a stoichiometric solution (*v*/*v*) of 0.1% naphthylethylenediamine dihydrochloride and 1% sulphanilamide. Equal volumes of culture supernatants and Griess reagent were mixed, and the absorbance was read at 550 nm using the FlexStation 3 multireader.

#### 2.4.3. Statistical Analysis

The in vitro assays were performed in triplicates, and the results were presented as the mean ± standard deviation (SD) of three independent experiments. The statistical significance was analyzed by Student’s *t*-test, and values of P less than 0.05 were considered significant.

## 3. Results and Discussion

### 3.1. Characterization of the Ruthenium Complexes

**Figure 3:**
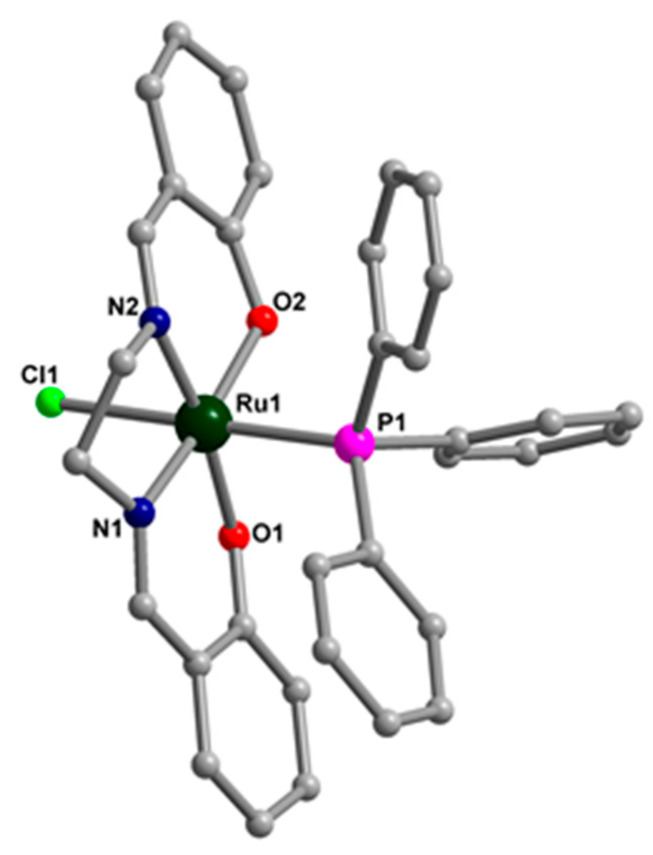
Crystal structure of the [Ru^III^(Salen)(PPh_3_)Cl] complex, along with atom numbering.

Only the RuSalen complex was obtained as single crystals suitable for X-ray diffraction, and its structure was confirmed by X-ray crystallography. Because the SCXRD investigation of the RuSalen complex was previously reported by Tang et al. [40] in 2018, herein we will briefly describe its crystal structure. [Ru(Salen)(PPh_3_)Cl] is a mononuclear Ru(III) complex that crystallizes in the P 2_1_/c monoclinic space group. Its structure consists of discrete neutral [Ru(Salen)(PPh_3_)Cl] units, as shown in Figure 3. In this structure, the ruthenium atom is six-coordinated by two phenoxido oxygen atoms [Ru1 − O1 = 2.023(4), Ru1 − O2 = 2.011(4) Å] and two imino nitrogen atoms [Ru1 − N1 = 1.987(5), Ru1 − N2 = 2.000(5) Å] from the tetradentate Schiff base ligand (H_2_Salen), in the equatorial plane, and by one PPh_3_ group [Ru1 − P1 = 2.349(2) Å] and one chloride atom [Ru1 − Cl1 = 2.4350(19) Å] into the axial positions, building a distorted octahedral environment around the Ru(III) center.

Electronic spectra of the Ru(III) and Ru(II) complexes have been recorded in the solid state in the 1000–200 nm range (spectra not shown). The UV–Vis spectra of all Ru(III) compounds show similar features and contain an intense broad band in the 200–1000 nm region, which is a multi-band coverage (three-structured absorption band in the ultraviolet and visible region, ~300, 400, 510, and 730 nm). The strong visible band in the range 500–1000 nm is due to the [Ru^III^N_2_O_2_PCl] chromophore (mainly charge-transfer transitions). In most of the Ru(III) complexes containing Schiff base ligands, charge-transfer transitions are prominent in the low-energy region, which obscures the weaker bands due to the *d*-*d* transition of the metal. It is therefore difficult to assign conclusively the bands of the ruthenium(III) complexes that appear in the visible region. The spectral profiles below 400 nm correspond to intra-ligand transitions (π-π* and n-π*) [33,41,42].

The magnetic moments, at room temperature, of all of the complexes, RuSalen, RuSalpnol, RuSalfen, and RuSaldiam, show that they are one-electron paramagnetic, confirming a low-spin d^5^, ^5^t_2g_ configuration for the ruthenium(III) ion (1.6 for RuSalen, 1.75 for RuSalpnol, 1.87 for RuSalfen, and 1.91 BM for RuSaldiam) [33,43,44]. The values of magnetic moments, close to expected for the spin-only value of a single unpaired electron species (1.73 BM), confirmed the (+3) state of ruthenium in these coordination compounds.

The absorption spectra of the RuSaldmen, RuSalampy, and RuSalaepy complexes are dominated in the visible region by absorption between 433 and 630 nm and in the UV region between 293 and 332 nm. The bands in the visible region are assigned to charge-transfer transitions (MLCT) and in the UV region to ligand (π-π* and n-π*) transitions [45]. The experimental magnetic susceptibilities at room temperature of the RuSaldmen, RuSalampy, and RuSalaepy complexes were negative, indicating that these compounds are diamagnetic, with the ruthenium ion being in the (+2) oxidation state.

### 3.2. Characterization of SBA–15 Functionalized with Ruthenium Complexes

**Figure 4:**
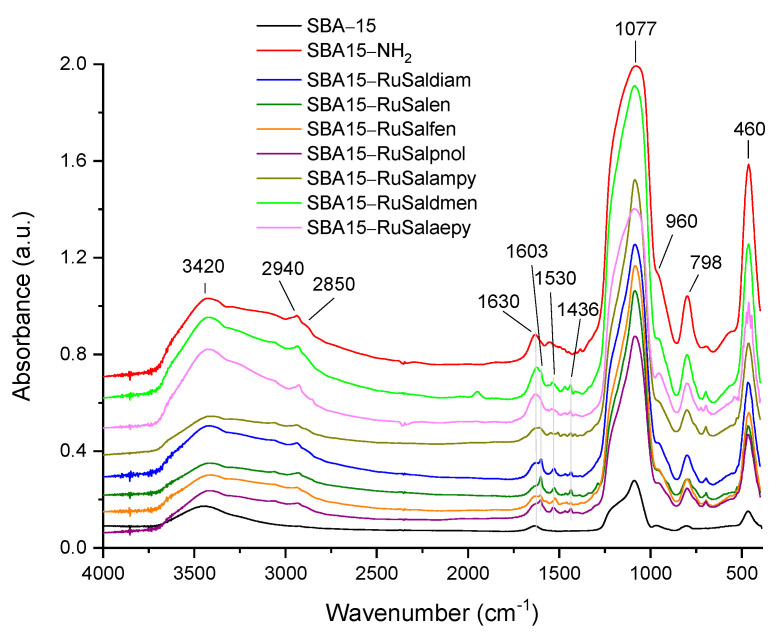
FT-IR spectra of SBA−15, SBA15−NH_2_ and SBA−15 functionalized with ruthenium complexes.

FT-IR spectra of SBA–15 and SBA–15 functionalized with ruthenium complexes are shown in Figure 4. The peaks located at 460 cm^−1^ (Si–O bending vibration), 798 cm^−1^ (symmetric Si–O–Si stretching vibration), 960 cm^−1^ (Si–OH stretching vibration), and 1077 cm^−1^ (asymmetric Si–O–Si stretching vibration) represent the fingerprint of silica framework in all materials [46]. The pair of bands in the interval 2850–2940 cm^−1^, characteristic of symmetric and asymmetric stretching aliphatic C–H bonds [47], can be observed in the spectra of SBA15–NH_2_ and all the samples functionalized with ruthenium complexes. The bands at 3420 and 1630 cm^−1^ are assigned to O–H bond stretching and bending vibrations of the silanol groups of the materials and the adsorbed H_2_O molecules. The new band at ~1553 cm^−1^ in the spectrum of SBA15–NH_2_, attributable to the bending vibration mode of N−H, confirms the grafting of aminopropyl groups on the surface of mesoporous silica. New bands of low intensity can be distinguished after functionalization of SBA–15 with ruthenium complexes, these bands being associated with the functional groups of the complexes. The most intense one, located at ~1603 cm^−1^, is attributed to the imine (C=N) stretching vibration of the Schiff bases in the structure of ruthenium complexes. This characteristic band of the ruthenium complexes confirms their presence in the mesoporous silica channels. The other bands of lower intensity, at around 1530 and 1436 cm^−1^ are assigned to C–N and C–C stretching vibrations and arise also from the attached ruthenium complex [33].

**Figure 5:**
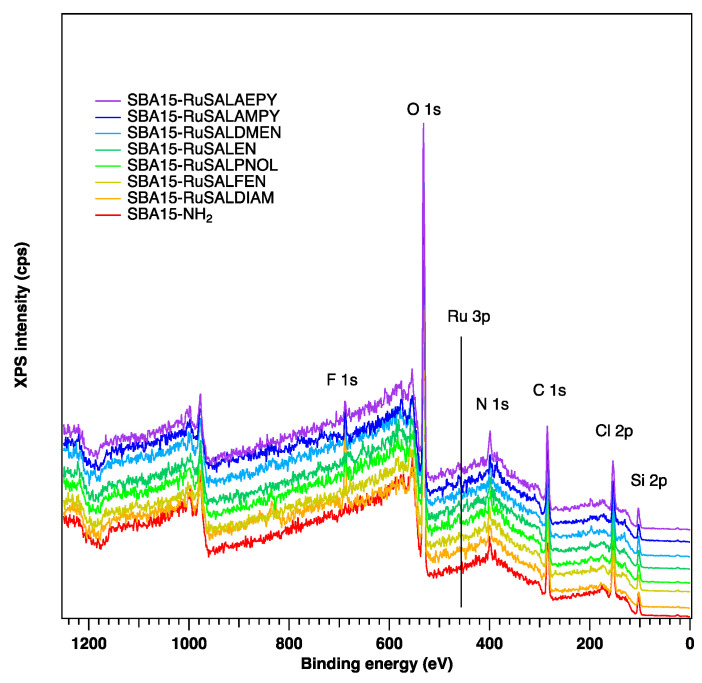
Survey XP spectra of the samples.

XPS analysis was conducted to obtain a more particular knowledge of the valence states of elements and the chemical composition of the samples. XPS spectra shown in Figure 5 confirm that, in all samples loaded with ruthenium complexes, ruthenium was successfully deposited and is present in the range between 0.2–0.4% on the surface of SBA–15. The C1s core level was fitted with five components: the first component at lower binding energies (~279.9 eV), corresponding to the Ru-C bond; the second component at 283.2 eV, associated with C–Si–O bonds; the third one at 284.6 eV, corresponding to the C–C/C=C bond; the fourth at 285.7 eV, corresponding to the C–N/C–O bonds; and the fifth at 287.0 eV, associated with C=O bonds (Figure S1). During modification with ruthenium complexes, an increase in the C–N component can be observed, which was expected since in the Ru complexes the carbon–nitrogen bond is present. The Si 2p core level presents three components: a component at low binding energies of 101.7 eV associated with the Si–C bond, the Si–O bond at 103.3 eV, and a component at higher binding energies of 104.5 eV, most probably due to some hydroxylated Si on the surface (Figure S1). The O1s core level presents in all samples three components: the first one at 531.2 eV assigned to the C-O bond, the second one at 532.6 eV associated with the Si-O component, and the third component corresponding to –OH groups at higher binding energies (533.7 eV) (Figure S1). The nitrogen is present in all samples in a relatively small amount (between 1.5 and 2.1%) and in all cases there are three components associated with imine N (398.2 eV), primary N (399.8 eV), and Ru–N at 401.3 eV (Figure S1). The XPS spectra highlight the presence of chlorine in all samples containing ruthenium (Figure S1), which suggests that the adsorption of the ruthenium complexes into the mesoporous silica channels is probably achieved through molecular interactions between the polar groups of the ruthenium complexes and the amino groups grafted onto the internal walls of SBA–15.

**Figure 6:**
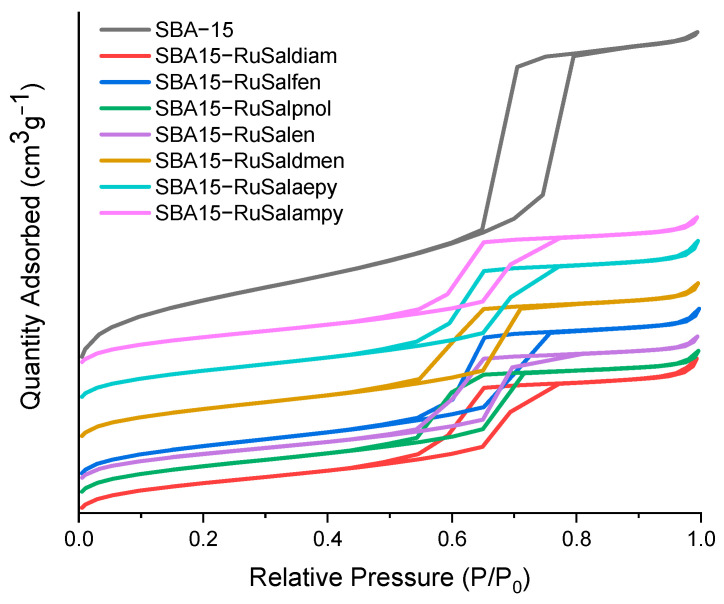
Nitrogen adsorption–desorption isotherms of the samples.

The textural analysis of the samples was performed by recording the N_2_ adsorption–desorption isotherms (Figure 6). Specific surface area, pore diameter, and total pore volume were determined from the sorption isotherms, and the results are listed in Table 1. According to the IUPAC classification [48], the nitrogen adsorption–desorption analysis indicated type IV isotherms for all samples, accompanied by type H1 hysteresis loops, characteristic for mesoporous materials with uniform cylindrical pores (Figure 6). After functionalization of SBA–15 with aminopropyl groups, a significant decrease in surface area (about 46%) and in total pore volume (about 40%) was observed, as well as a corresponding decrease in pore diameter (Table 1). A higher decrease in the values of surface area (58–63%) and total pore volume (52–58%) compared to the corresponding ones for SBA–15 was observed after the immobilization of ruthenium complexes. A decrease of 10–17% was also observed in the average pore size. These findings suggest a uniform immobilization of the ruthenium complexes onto the internal pore walls of SBA–15, resulting in reduced accessible space for adsorbed nitrogen.

Zeta (ζ)-potential measurements were carried out to analyze the net surface charge of the samples, a very important parameter on which the internalization of nanoparticles by cancer cells depends [49]. The obtained values are shown in Table 1. The negative ζ-potential of pristine SBA–15 (−24.7 mV) is due to the presence of silanol groups. Functionalization of SBA–15 with aminopropyl groups led to a positive ζ-potential (+24.4 mV), while loading with ruthenium complexes further increased the ζ-potential of the obtained hybrid materials. Since all the ruthenium-loaded materials have positive and relatively high ζ-potential values, they can be expected to target cancer cells efficiently due to electrostatic attraction to their negatively charged membrane [50]. These ζ-potential values also reveal a relatively good colloidal stability of mesoporous silica loaded with Ru(II)/Ru(III) complexes in aqueous medium.

Bearing in mind that these compounds were synthesized in order to study their biological activity (antibacterial and cytotoxic activity), it is very important to know their stability in solution. For this purpose, a spectroscopic study was carried out using the UV-Vis technique, on the compounds suspended in deionized water (250 μg/mL), at 37 °C (Figure S2). No significant spectral changes in the studied materials were observed after 24 and 72 h, respectively, after the preparation of the suspensions, which shows their very good stability in the aqueous environment. Therefore, it can be said that the studied hybrid systems based on mesoporous silica functionalized with Ru(II) and Ru(III) complexes can be used in aqueous suspensions to determine their biological activity.

**Figure 7:**
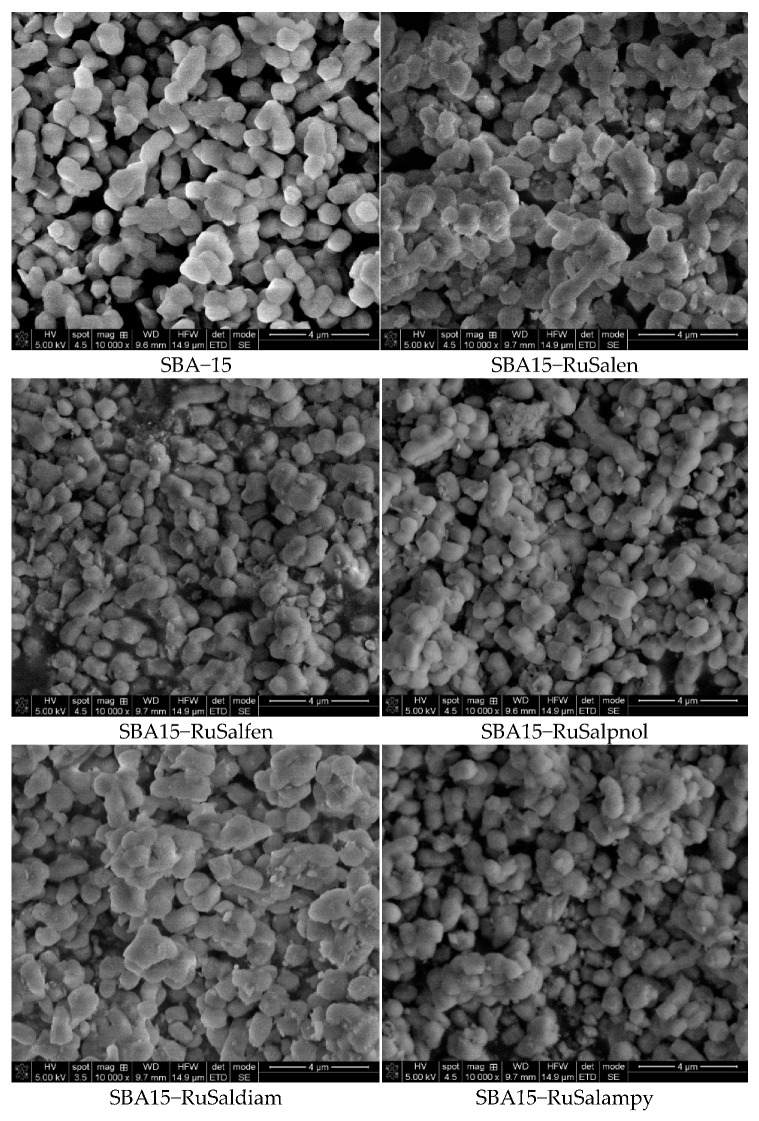

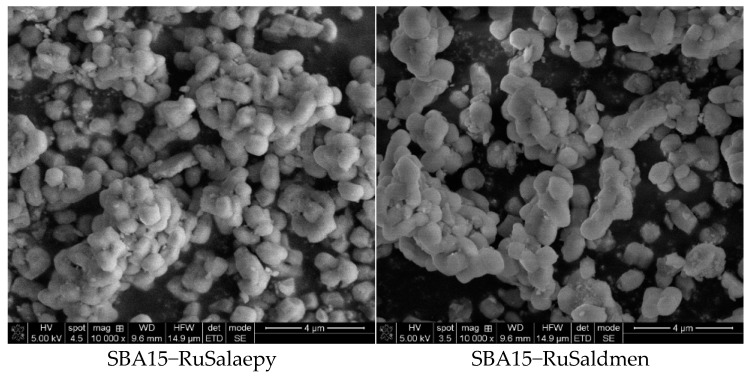
SEM images of the investigated samples.

The morphologic characterization of the samples was performed by SEM, and the acquired images are shown in Figure 7. Pure SBA–15 consists of short-rod-like particles with typical wheat-like morphologies and relatively uniform sizes ranging between 0.5 and 1.0 μm. For all the ruthenium-containing mesoporous materials, no significant changes were observed in particle sizes and shapes. This suggests that loading with ruthenium complexes does not affect the macroscopic morphology of the materials.

**Figure 8:**
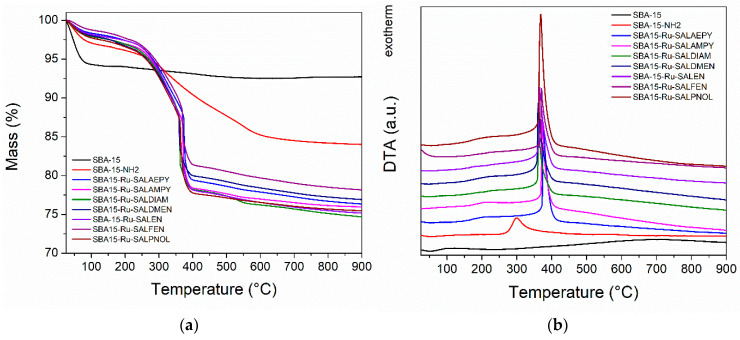
Thermogravimetric (**a**) and differential thermal analyses (**b**) of the SBA–15, SBA15–NH_2_, and SBA–15 functionalized with ruthenium complexes.

Thermogravimetric analyses coupled with differential thermal analyses were carried out in order to evidence the combustion of the functionalized organic groups. All samples exhibit variable mass loss from 25 to 110 °C, (Figure 8a) accompanied by an endothermic thermal effect (Figure 8b), which is likely caused by physisorbed water evaporation. SBA–15 shows a gradual mass loss above 200 °C, explained by the condensation of surface silanol groups. The combustion of aminopropyl groups can be noticed for SBA15–NH_2_ as a mass loss event between 250 and 650 °C, accompanied by an exothermic thermal effect. The samples containing the Ru complexes all exhibit similar mass loss effects between 200 and 500 °C, caused by the superposition of the combustion of organic ligand and silica functional groups. The composition of the samples was computed assuming that the silanol content of SBA–15 and the aminopropyl content of SBA15–NH_2_ are persevered for all other materials (Table 2). The SBA15–NH_2_ matrix contains 11.7% wt. aminopropyl groups with respect to the dry sample mass. The materials containing the Ru complexes exhibit 8.4–11.6% weight loss associated with the ligand combustion. Thus, TGA analyses show the successful functionalization of SBA–15 and the incorporation of the complexes.

### 3.3. Biological Evaluation

#### 3.3.1. Antimicrobial Activity

The qualitative evaluation of the functionalized mesoporous silica samples revealed that, except for SBA–15, all of the samples exhibited an inhibitory effect on the growth of Gram-positive strains (*S. aureus* and *E. faecalis*), the largest growth inhibition diameters being recorded for SBA15–RuSalaepy, SBA15–RuSalen, and SBA15–RuSaldiam (Table 3). SBA–15 had no effect on any of the strains tested. The Ru(II)- and Ru(III)-based compounds inhibited to a lesser extent the development of the Gram-negative *E. coli* strain when tested on solid media, while the *P. aeruginosa* growth was not impaired by any of the tested compounds (Table 3).

**Figure 9:**
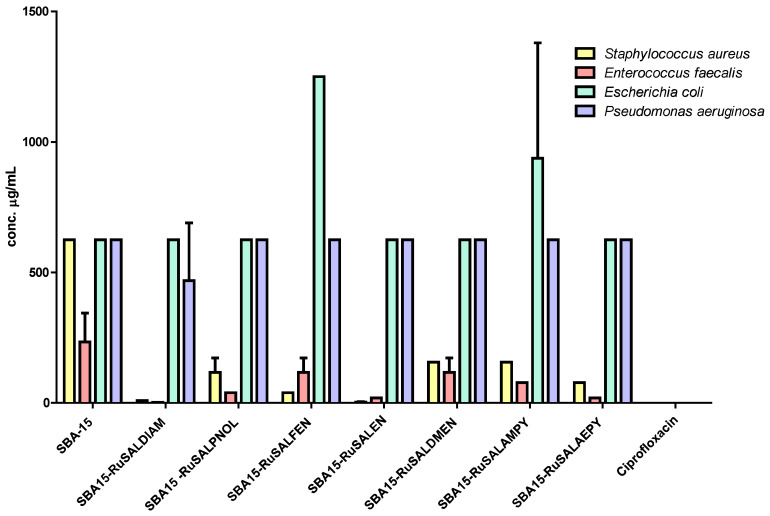
Minimal inhibitory concentration (MIC) values of the investigated mesoporous silica functionalized with Ru(II) and Ru(III) complexes.

The quantitative evaluation of the antimicrobial activity confirmed the inhibitory effects of the mesoporous silica functionalized with Ru(II) and Ru(III) complexes, especially against the Gram-positive tested strains. Except for SBA–15, all of the other compounds had MIC values of maximum 156 μg/mL for the Gram-positive strains (Figure 9, Table 4), with SBA15–RuSaldiam and SBA15–RuSalen being the most active. Regarding the Gram-negative strains, most of the MIC values were higher (625 μg/mL for all samples, except for SBA15–RuSalfen with an MIC of 1250 μg/mL against *E. coli*) (Figure 9, Table 4).

**Figure 10:**
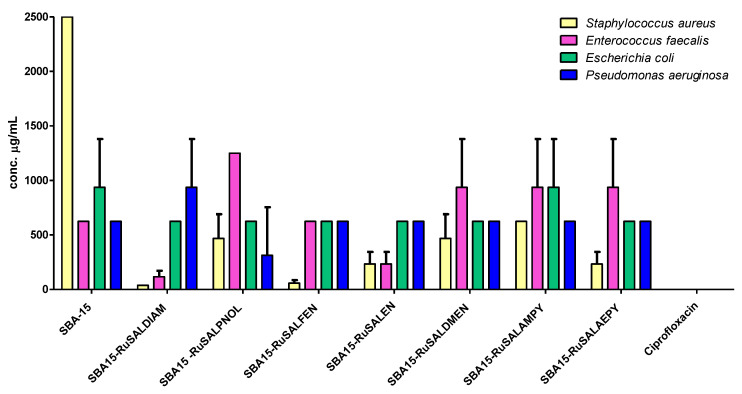
Minimum inhibitory concentrations of the bacterial adherence to inert substrata of the mesoporous silica functionalized with Ru(II) and Ru(III) complexes.

The inhibition of bacterial adherence to the inert substrata was observed for most of the mesoporous silica compounds at a concentration of 625 μg/mL. SBA15–RuSaldiam, SBA15–RuSalen were the most active against *S. aureus* and *E. faecalis* strains, and SBA15–RuSalfen against the *S. aureus* strain (Figure 10, Table 5).

Our results confirm the fact that the ruthenium-based compounds have good antimicrobial activity towards Gram-positive bacteria and, to a lesser extent, to Gram-negative species, with SBA15–RuSaldiam and SBA15–RuSalen being the most efficient against the tested bacterial strains.

#### 3.3.2. Cytotoxicity Evaluation

**Figure 11:**
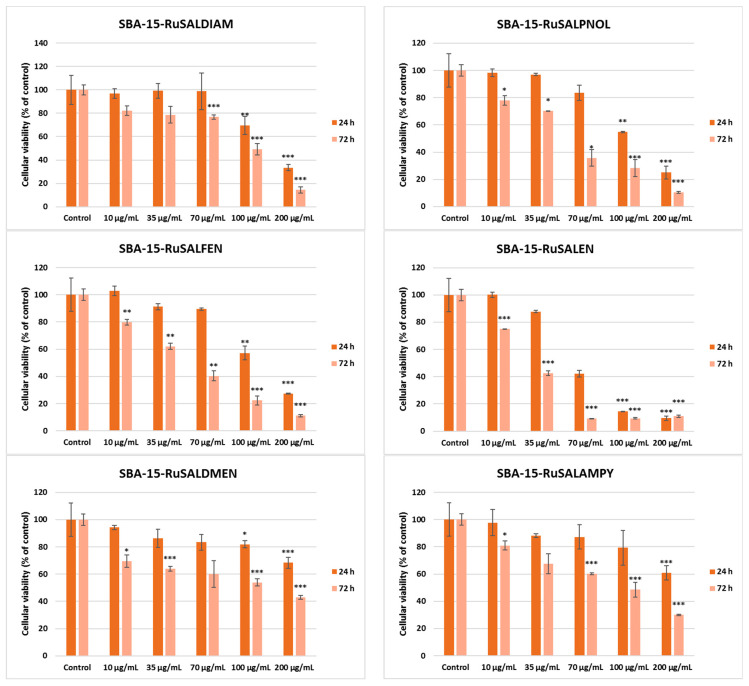

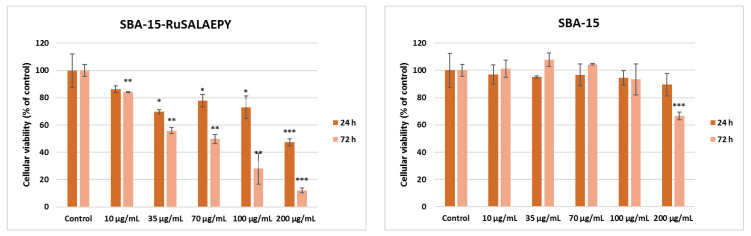
Effect of different concentrations of Ru-containing hybrid materials (0, 10, 35, 70, 100, and 200 μg/mL) on viability of A549 lung tumor cells after 24 and 72 h of exposure, evaluated by MTT assay. Results are presented as mean values ± SD relative to control (n = 3) (*** *p* < 0.001, ** *p* < 0.01 and * *p* < 0.05 compared to control).

The in vitro studies (Figure 11) were carried out to assess the viability of A549 lung tumor cells after 24 and 72 h of exposure to various concentrations (0–200 μg/mL) of Ru-based hybrid materials, as well as their potential to induce inflammation. The cellular viability assay revealed that all ruthenium complex-containing samples inhibited cell growth in a time- and dose-dependent manner compared to the control (Figure 11). In contrast, SBA–15 did not show the same pattern of viability decrease at the highest concentration tested (200 μg/mL) reducing the number of live cells only by 10% of the control after 24 h. Among the investigated materials, those containing Ru(III) complexes with compartmental ligands (SBA15–RuSalpnol, SBA15–RuSalen, SBA15–RuSaldiam, and SBA15–RuSalfen) showed a higher cytotoxic activity on A549 lung tumor cells than those with open ligands (SBA15–RuSaldmen, SBA15–RuSalampy, and SBA15–RuSalaepy), regardless of the incubation time. It is worth mentioning that SBA15–RuSalen showed the highest cytotoxic potential, diminishing the viable cell population by half of the control at a concentration of 70 μg/mL after 24 h of incubation, and at 35 μg/mL after 72 h of incubation. Furthermore, higher concentrations of this compound reduced the viability of A549 cells by more than 90% of the control.

Ruthenium-based systems have gained great attention recently for their activity against cancer [50]. Previously, it was shown that ruthenium-loaded palmitoyl ascorbate (PA)-modified mesoporous silica was able to inhibit cancer cell growth and induce their apoptosis through superoxide generation and DNA damage [31]. Our results confirmed the good biological activity of Ru-based hybrid materials against cancer cell growth, inducing their death, most probably by oxidative stress activation. In addition, we noticed a higher potency of Ru(III) complexes than Ru(II) ones. Previous reports showed that Ru(II) complexes are more reactive than Ru(III) [51], but less cytotoxic [52]. These could interact with the thiol groups in the cell, modulating the activity of intracellular enzymes and signaling pathways.

**Figure 12:**
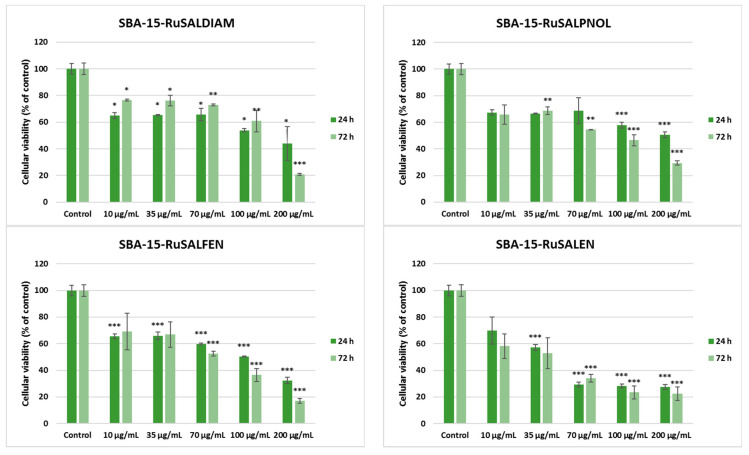

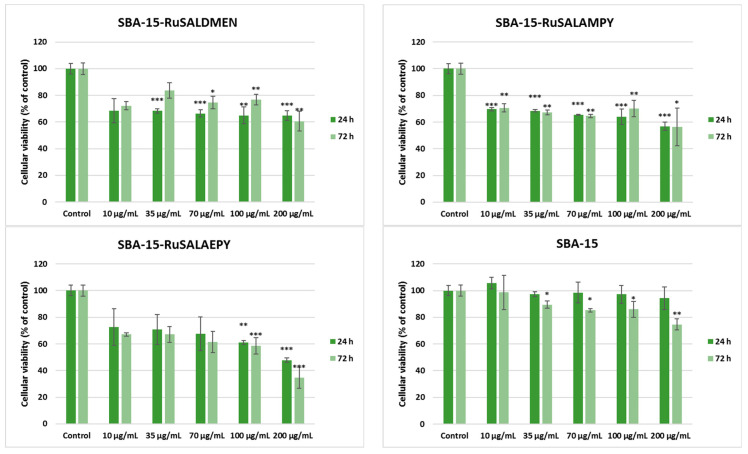
Effect of different concentrations of Ru-containing hybrid materials (0, 10, 35, 70, 100, and 200 μg/mL) on viability of MRC-5 non-tumoral lung fibroblasts after 24 and 72 h of exposure, evaluated by MTT assay. Results are presented as mean values ± SD relative to control (n = 3) (*** *p* < 0.001, ** *p* < 0.01 and * *p* < 0.05 compared to control).

In order to check if these Ru-containing hybrid materials could affect the viability of non-tumoral cells, the MTT assay was performed also after 24 and 72 h of incubation with normal lung fibroblasts MRC-5 (Figure 12). A decrease in viable cell number was observed compared to the control after both periods of exposure for all types of materials tested, and no great difference was noticed between values obtained after 24 h and those after 72 h. The highest reduction in cell viability was determined after incubation with concentrations higher than 70 μg/mL of SBA–15–RuSaldiam, SBA–15–RuSalpnol, SBA–15–RuSalfen, and SBA–15–RuSalen. However, it is important to highlight that the viability percentages for non-tumor cells were higher compared to the values obtained in the case of A549 cancer cells after 72 h. These findings could suggest that MRC-5 cells were more sensitive to the compounds tested after the first day of exposure, the values being lower than those recorded for A549 epithelial cells, but after another 2 days, the tumor cells were much more affected, especially at high concentrations. This could confirm that despite the cytotoxicity exerted on normal cells, the ruthenium-containing hybrid materials possess a good anti-cancer potential.

The IC50 values (Table 6) obtained from cell survival plots (Figures S3–S6) showed that the SBA–15–RuSalen compound has the most potent antitumor efficiency, with a concentration of 23.9 μg/mL being able to inhibit half of A549 cells’ growth compared to control after 72 h. The selectivity index (the ratio between the IC_50_ for normal cell line and IC_50_ for the cancer cell line) for this compound was 2.4, proving the higher toxicity against tumor cells than against normal ones. By comparing the result of SBA–15–RuSalen with a positive control, such as cisplatin, the standard therapy for patients with lung cancer [53], we observed an almost-similar IC_50_ value (26 ± 3.0 μg/mL), as it was previously reported [54] after 72 h of incubation of this drug with A549 cells. Furthermore, it is important to highlight that all Ru-containing hybrid materials exhibited lower IC_50_ values compared to the SBA–15 compound.

**Figure 13:**
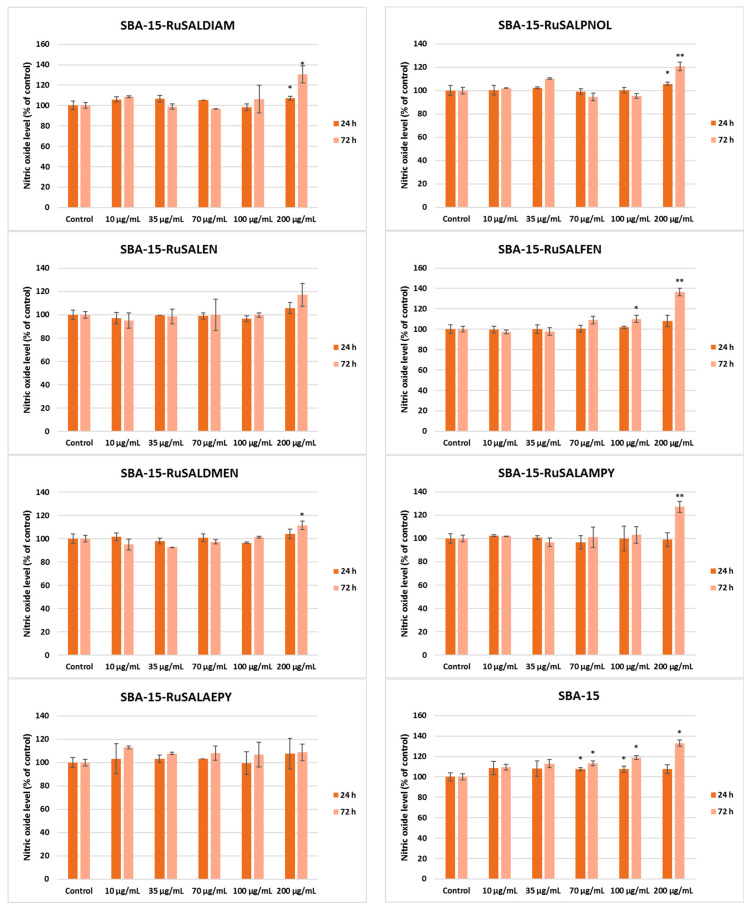
Effect of different concentrations of Ru-containing hybrid materials (0, 10, 35, 70, 100, and 200 μg/mL) on the NO level released by A549 tumor lung cells after 24 and 72 h of exposure, evaluated by the Griess assay. Results are presented as mean values ± SD relative to control (n = 3) (** *p* < 0.01 and * *p* < 0.05 compared to control).

NO is toxic to cells in high concentrations, and measuring its release in cell culture media can provide a valuable way to assess the toxic effects of nanoparticles, materials, drugs, or other compounds on cells [55]. This molecule is also involved in the inflammatory response, and quantifying its release can indicate the level of inflammation in the cells. The results of the Griess assay showed an increase in the NO release compared to the control only after 72 h of incubation with the highest concentration used (200 μg/mL) (Figure 13). This could indicate that inflammation and high toxicity were induced only by the high quantity of compounds tested.

**Figure 14:**
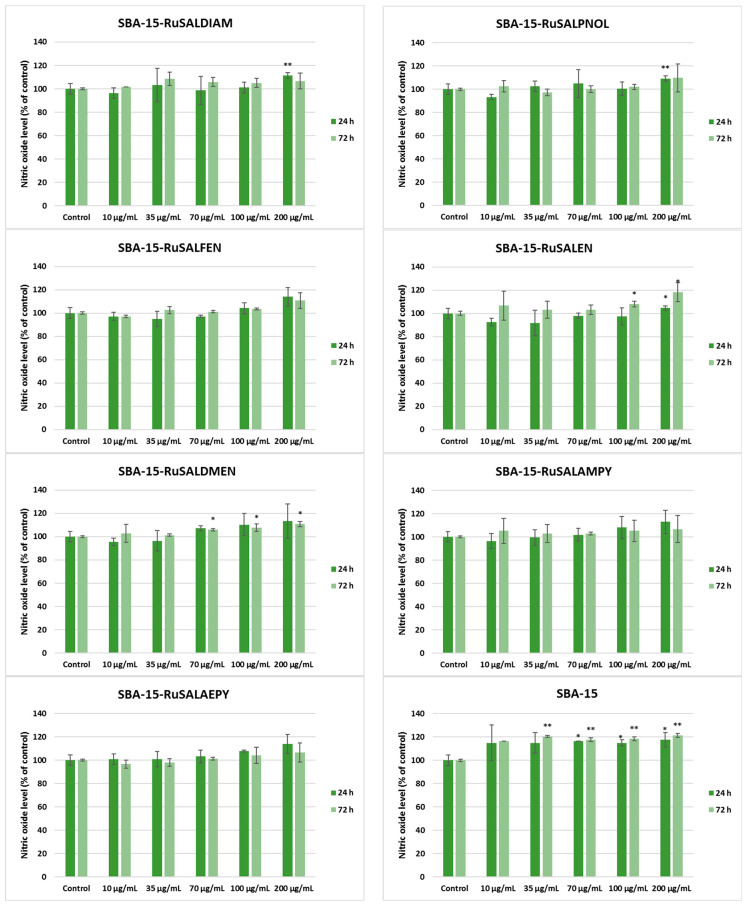
Effect of different concentrations of Ru-containing hybrid materials (0, 10, 35, 70, 100, and 200 μg/mL) on the NO level released by MRC-5 non-tumor lung fibroblasts after 24 and 72 h of exposure, evaluated by the Griess assay. Results are presented as mean values ± SD relative to control (n = 3) (** *p* < 0.01 and * *p* < 0.05 compared to control).

Regarding the effect on MRC-5 cells, Ru-containing hybrid materials induced an increase in NO release compared to the control only after the incubation with 200 μg/mL, but the values did not exceed those registered in the case of A549 cancer cells. The most elevated values were noticed in the case of SBA–15 (Figure 14).

## 4. Conclusions

In this work, we have obtained and characterized by various methods a new series of nanostructured materials based on SBA–15 mesoporous silica loaded with Ru(II) and Ru(III) complexes bearing Schiff base ligands derived from salicylaldehyde and various amines. Their antimicrobial activity was evaluated against *S. aureus*, *E. faecalis, E. coli,* and *P. aeruginosa*, while the anticancer activity was investigated in vitro against A549 lung tumor cells and MRC-5 normal lung fibroblasts. The results of the antibacterial activity suggest the promising potential of SBA15–RuSaldiam, SBA15–RuSalen, and SBA15–RuSalaepy for the development of novel antibacterial drugs, efficient against *S. aureus* and *E. faecalis* Gram-positive strains, two of the most fearful resistant opportunistic nosocomial pathogens, both in planktonic and adherent growth states. The compounds SBA15–RuSalpnol, SBA15–RuSalen, SBA15–RuSaldiam, and SBA15–RuSalfen proved to have the highest cytotoxic potential demonstrated on the A549 tumor cells. Of these, SBA15–RuSalen stands out as the most potent, with an IC_50_ index of 23.9 μg/mL and a selectivity index of 2.4. Thus, these activities open the avenue for the development of multi-pharmacologically active compounds with antiproliferative activity against prokaryotic and eukaryotic cells.

## Figures and Tables

**Table 1:** Textural parameters (S_BET_, total pore volume, average pore size) of the samples.

Sample	S_BET_ (m^2^g^−1^)	Pore Volume (cm^3^g^−1^)	Average Pore Size (nm)	Zeta Potential (mV)
SBA−15	778.8	1.177	6.04	−24.7
SBA15−NH_2_	417.5	0.700	5.37	+24.4
SBA15−RuSaldiam	292.0	0.508	5.35	+28.6
SBA15−RuSalfen	324.1	0.562	5.39	+32.9
SBA15−RuSalpnol	305.2	0.485	4.98	+30.8
SBA15−RuSalen	284.7	0.484	5.22	+29.1
SBA15−RuSaldmen	321.7	0.524	5.10	+42.7
SBA15−RuSalampy	297.8	0.497	5.26	+35.4
SBA15−RuSalaepy	309.1	0.532	5.35	+30.2

**Table 2:** Sample composition (silica, silanol, aminopropyl, and ligand) computed from TGA.

Sample	Silica (% wt.)	Silanol (% wt.)	Aminopropyl (% wt.)	Ligand (% wt.)
SBA−15	98.3	1.7		
SBA15−NH_2_	86.8	1.5	11.7	
SBA15−RuSalaepy	78.1	1.3	10.5	10.1
SBA15−RuSalampy	77.8	1.3	10.5	10.4
SBA15−RuSaldmen	78.9	1.3	10.6	9.2
SBA15−RuSaldiam	76.8	1.3	10.3	11.6
SBA15−RuSalen	77.0	1.3	10.4	11.4
SBA15−RuSalfen	79.6	1.3	10.7	8.4
SBA15−RuSalpnol	77.5	1.3	10.4	10.7

**Table 3:** Diameters of the growth inhibition zones (mm).

Sample	Strain			
	*S. aureus*	*E. faecalis*	*E. coli*	*P. aeruginosa*
SBA−15	-	-	-	-
SBA15−RuSaldiam	10	10	4	-
SBA15−RuSalpnol	7	6	4.5	-
SBA15−RuSalfen	6	6	5	-
SBA15−RuSalen	10.5	10	5	-
SBA15−RuSaldmen	6	5.5	5	-
SBA15−RuSalampy	7	7	5	-
SBA15−RuSalaepy	11	16	5	-
Ciprofloxacin	25	24	30	27

**Table 4:** Minimal inhibitory concentration (MIC) values of the investigated mesoporous silica functionalized with Ru(II) and Ru(III) complexes expressed as average in μg/mL.

Sample	Strain			
	*S. aureus*	*E. faecalis*	*E. coli*	*P. aeruginosa*
SBA−15	625	234	625	625
SBA15−RuSaldiam	9	2	625	468.5
SBA15−RuSalpnol	117	39	625	625
SBA15−RuSalfen	39	117	1250	625
SBA15−RuSalen	3	19	625	625
SBA15−RuSaldmen	156	117	625	625
SBA15−RuSalampy	156	78	937,5	625
SBA15−RuSalaepy	78	19	625	625
Ciprofloxacin	0.15	0.31	0.009	0.15

**Table 5:** Minimum inhibitory concentrations of the bacterial adherence to inert substrata of the mesoporous silica functionalized with Ru(II) and Ru(III) complexes expressed as average in μg/mL.

Sample	Strain			
	*S. aureus*	*E. faecalis*	*E. coli*	*P. aeruginosa*
SBA−15	2500	625	937.5	625
SBA15−RuSaldiam	39	117	625	937.5
SBA15−RuSalpnol	468.5	1250	625	313.12
SBA15−RuSalfen	58.5	625	625	625
SBA15−RuSalen	234	234	625	625
SBA15−RuSaldmen	468.5	937.5	625	625
SBA15−RuSalampy	625	937.5	937.5	625
SBA15−RuSalaepy	234	937.5	625	625
Ciprofloxacin	0.15	0.31	0.009	0.15

**Table 6:** IC_50_ values (μg/mL) obtained by MTT assay for Ru-containing hybrid materials after 24 and 72 h of incubation with A549 lung tumor cells. Data are expressed as means of three determinations.

Sample	IC_50_ (μg/mL)			
	A549 Lung Tumor Cells		MRC-5 Lung Non-Tumoral Cells	
	24 h	72 h	24 h	72 h
SBA−15	872.3	244.2	2386.1	916.9
SBA15−RuSaldiam	151.0	112.6	144.1	134.9
SBA15−RuSalpnol	118.9	56.0	218.3	76.8
SBA15−RuSalfen	126.1	48.1	92.4	68.5
SBA15−RuSalen	62.6	23.9	53.0	57.8
SBA15−RuSaldmen	682.0	141.1	625.3	323.3
SBA15−RuSalampy	303.6	93.3	283.6	315.4
SBA15−RuSalaepy	219.0	50.3	284.0	126.4

## Data Availability

Not applicable.

## Supplementary Materials

The following supporting information can be downloaded at: https://www.mdpi.com/article/10.3390/pharmaceutics15051458/s1, Figure S1: C1s, O1s, Si2p, N1s, Ru3p, and Cl2p deconvoluted photoelectron spectra for the investigated samples; Figure S2: UV-Vis spectra of the investigated mesoporous silica functionalized with Ru(II) and Ru(III) complexes in aqueous solution (250 μg/mL): (a) immediately after preparation; (b) after 24 h; (c) after 72 h; Figure S3: Cell survival graphs (%) obtained by MTT assay for Ru-containing hybrid materials after 24 h of incubation with A549 lung tumor cells; Figure S4: Cell survival graphs (%) obtained by MTT assay for Ru-containing hybrid materials after 72 h of incubation with A549 lung tumor cells; Figure S5: Cell survival graphs (%) obtained by MTT assay for Ru-containing hybrid materials after 24 h of incubation with MRC-5 lung non-tumoral cells; Figure S6: Cell survival graphs (%) obtained by MTT assay for Ru-containing hybrid materials after 72 h of incubation with MRC-5 lung non-tumoral cells.
