# Supplementary material for: New Nanostructured Materials Based on Mesoporous Silica Loaded with Ru(II)/Ru(III) Complexes with Anticancer and Antimicrobial Properties
Source: Pharmaceutics. 2023 May 10;15(5):1458. doi: 10.3390/pharmaceutics15051458 (PMC10221598; doi:10.3390/pharmaceutics15051458)
Supplement: Supplementary file 1 [file pharmaceutics-15-01458-s001.zip › pharmaceutics-2317121-SI.pdf]

pharmaceutics

MDPI

Supplementary Materials: New nanostructured materials based on mesoporous silica loaded with Ru(II)/Ru(III) complexes with anticancer and antimicrobial properties

Gabriela Marinescu, Daniela C. Culita, Teodora Mocanu, Raul-Augustin Mitran, Simona Petrescu, Miruna S. Stan, Mariana C. Chifiriuc and Marcela Popa

Electronic Supplementary Information:

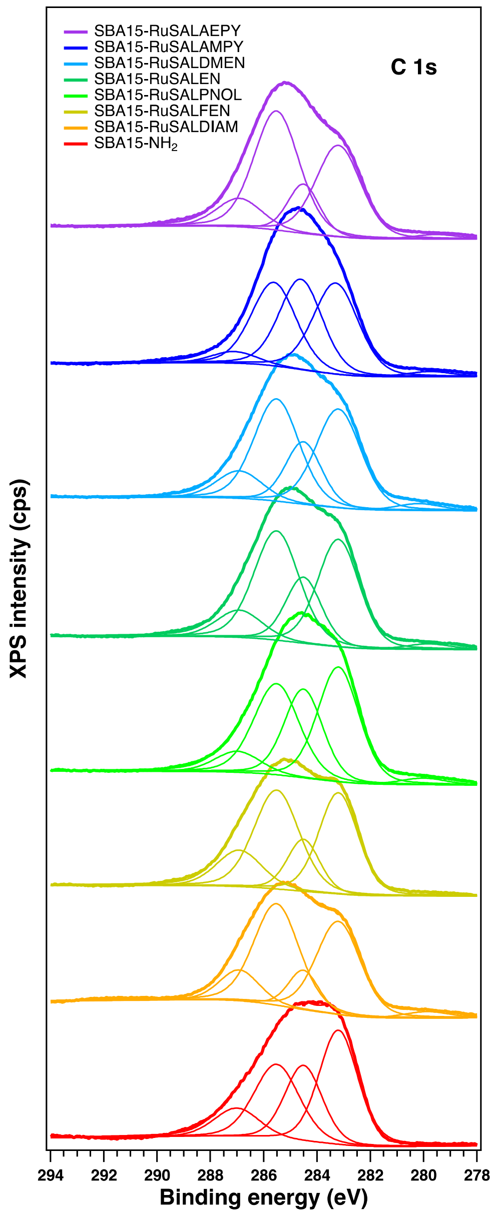

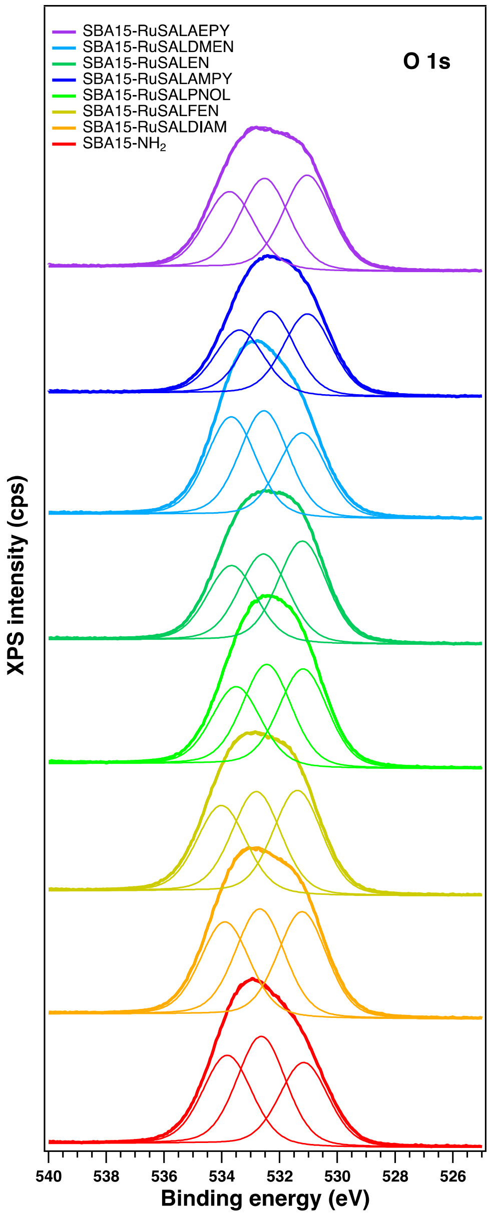

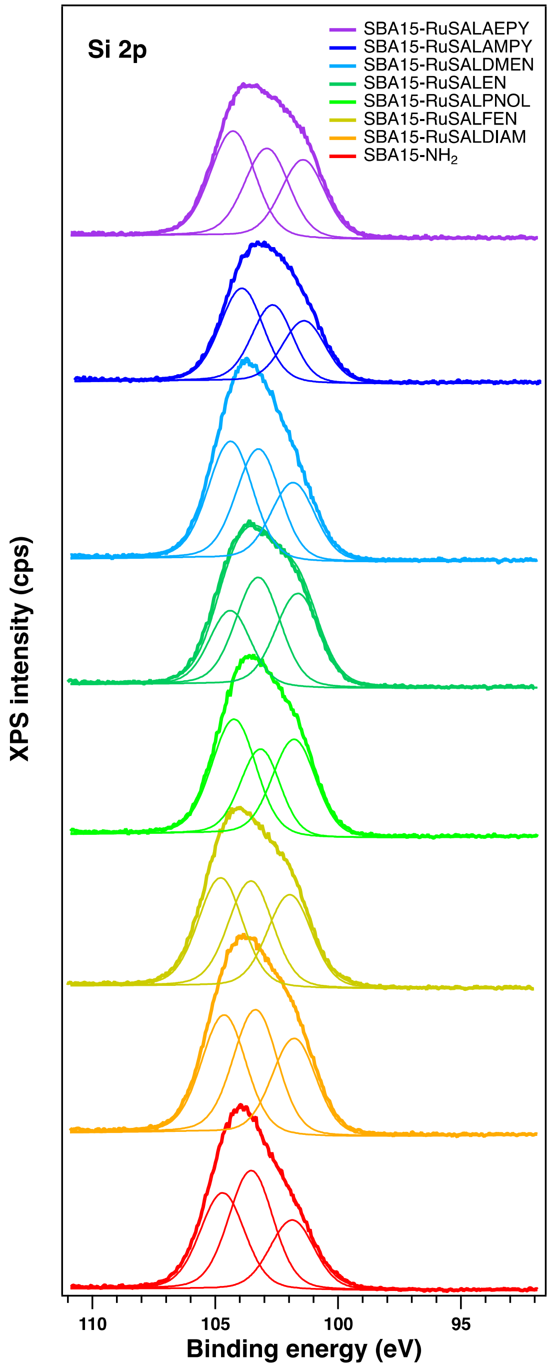
Si 2p
SBA15-RuSALAEPY
SBA15-RuSALAMPY
SBA15-RuSALDMEN
SBA15-RuSALEN
SBA15-RuSALPNOL
SBA15-RuSALFEN
SBA15-RuSALDIAM
SBA15-NH$_2$
XPS intensity (cps)
110
105
100
95
Binding energy (eV)

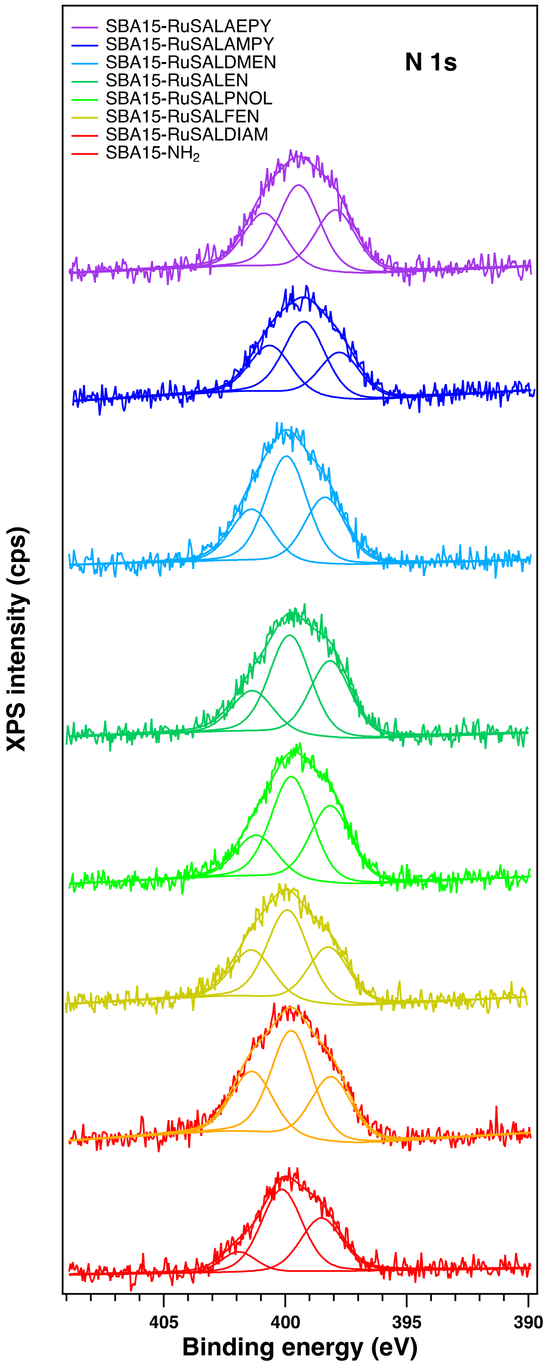
N 1s
SBA15-RuSALAEPY
SBA15-RuSALAMPY
SBA15-RuSALDMEN
SBA15-RuSALEN
SBA15-RuSALPNOL
SBA15-RuSALFEN
SBA15-RuSALDIAM
SBA15-NH$_2$
XPS intensity (cps)
405
400
395
390
Binding energy (eV)

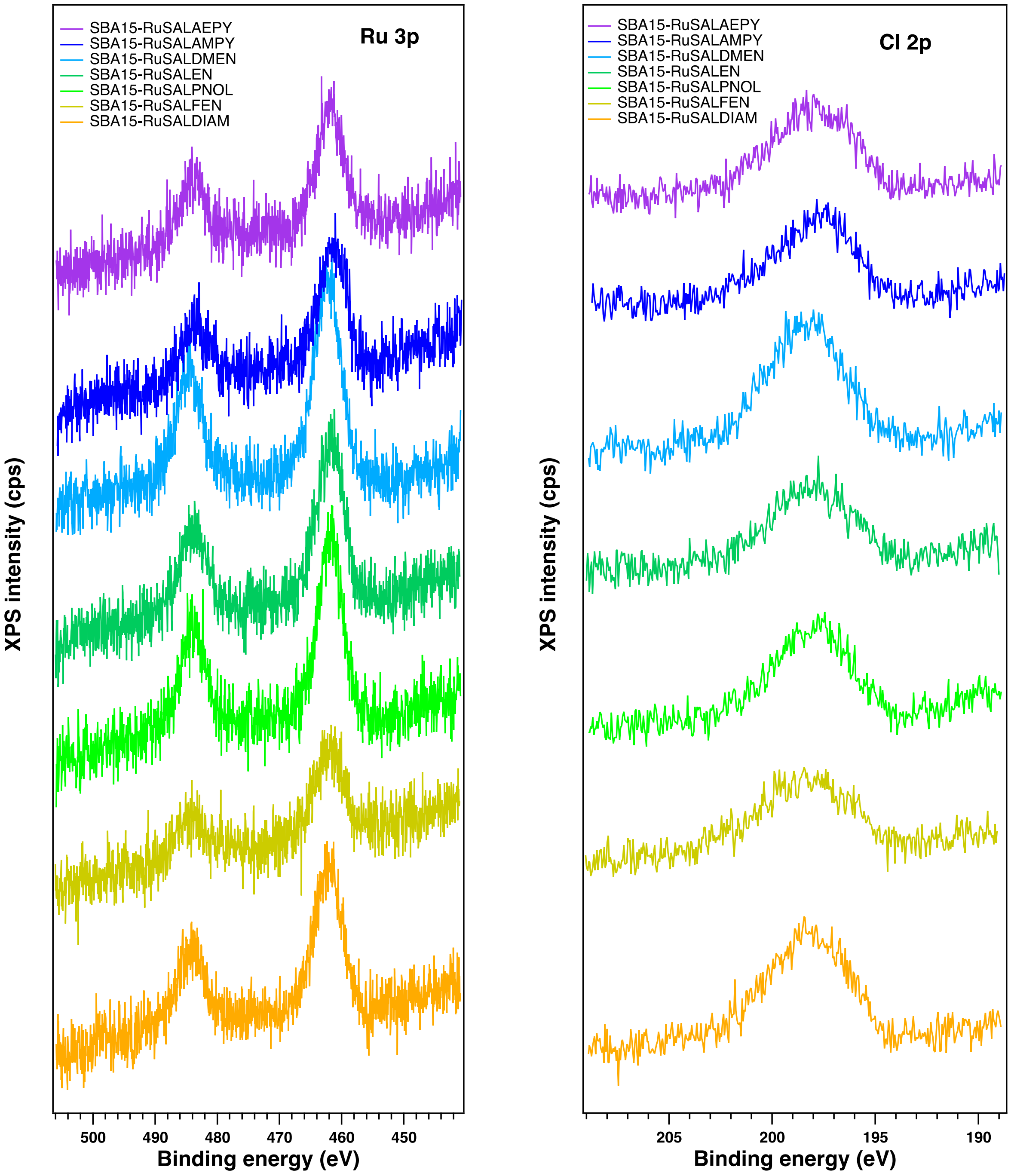

Figure S1. C 1s, O 1s, Si 2p, N 1s, Ru 3p, and Cl 2p deconvoluted photoelectron spectra for the investigated samples.

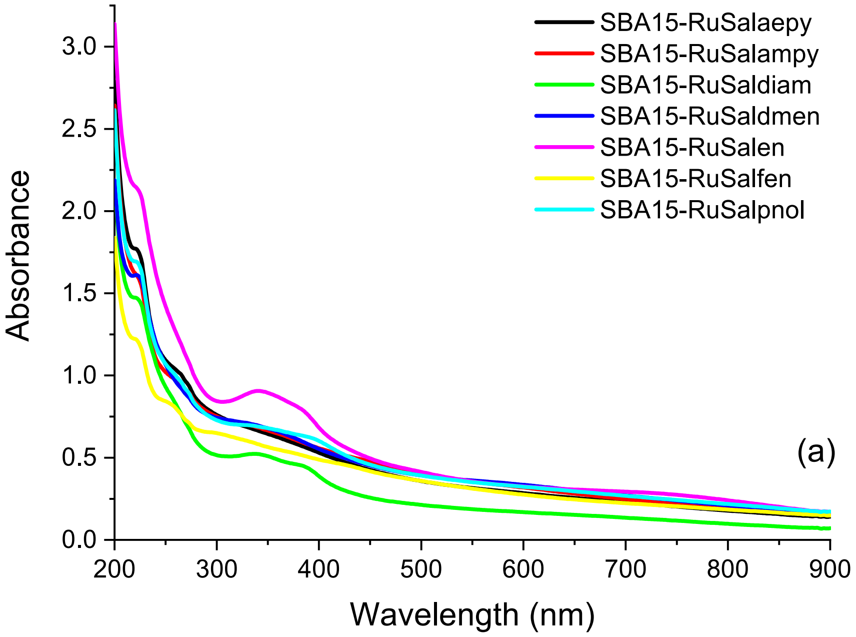
SBA15-RuSalaepy
SBA15-RuSalampy
SBA15-RuSaldiam
SBA15-RuSaldmen
SBA15-RuSalen
SBA15-RuSalfen
SBA15-RuSalpnol
Absorbance
Wavelength (nm)
(a)

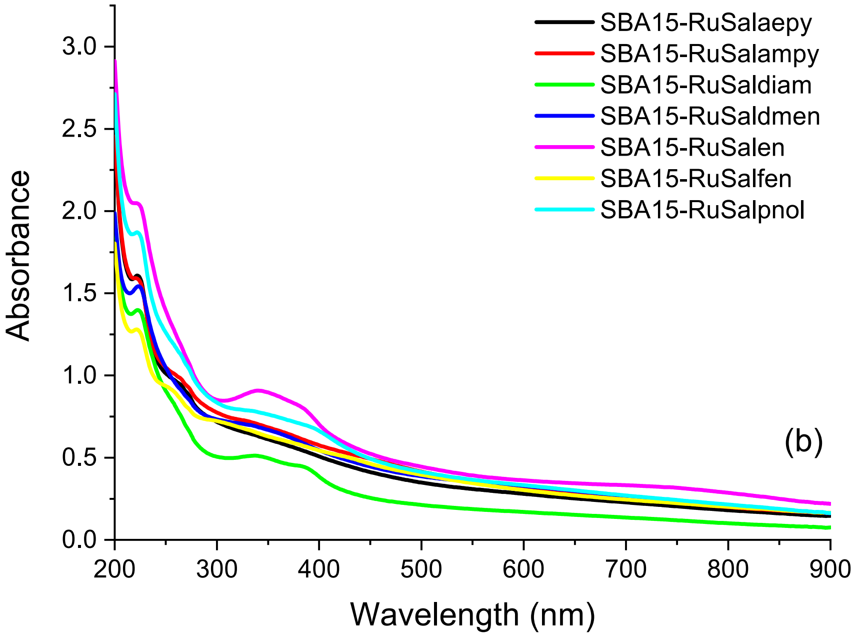
SBA15-RuSalaepy
SBA15-RuSalampy
SBA15-RuSaldiam
SBA15-RuSaldmen
SBA15-RuSalen
SBA15-RuSalfen
SBA15-RuSalpnol
Absorbance
Wavelength (nm)
(b)

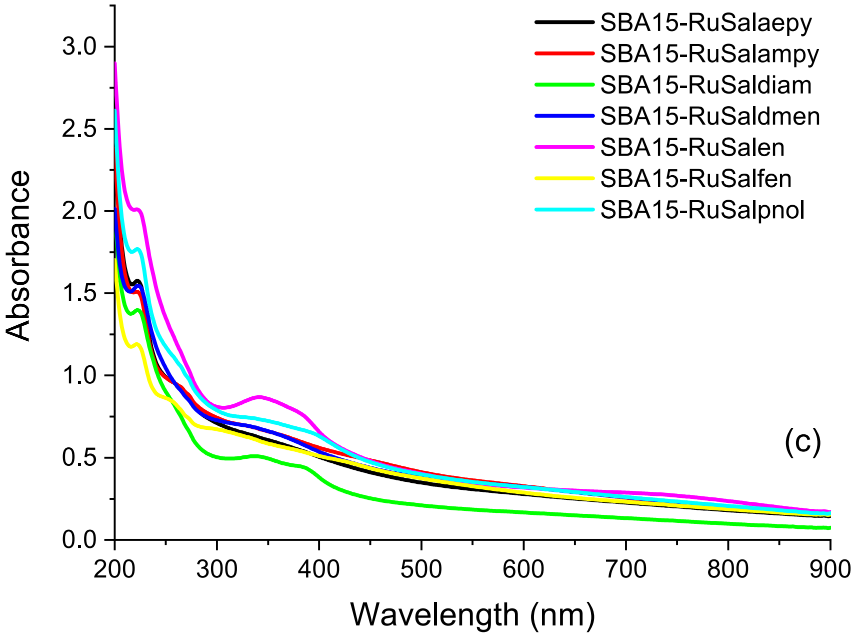

Figure S2. UV-Vis spectra of the investigated mesoporous silica functionalized with Ru(II) and Ru(III) complexes in aqueous solution (250 µg/ml): (a) immediately after preparation; (b) after 24 h; (c) after 72 h.

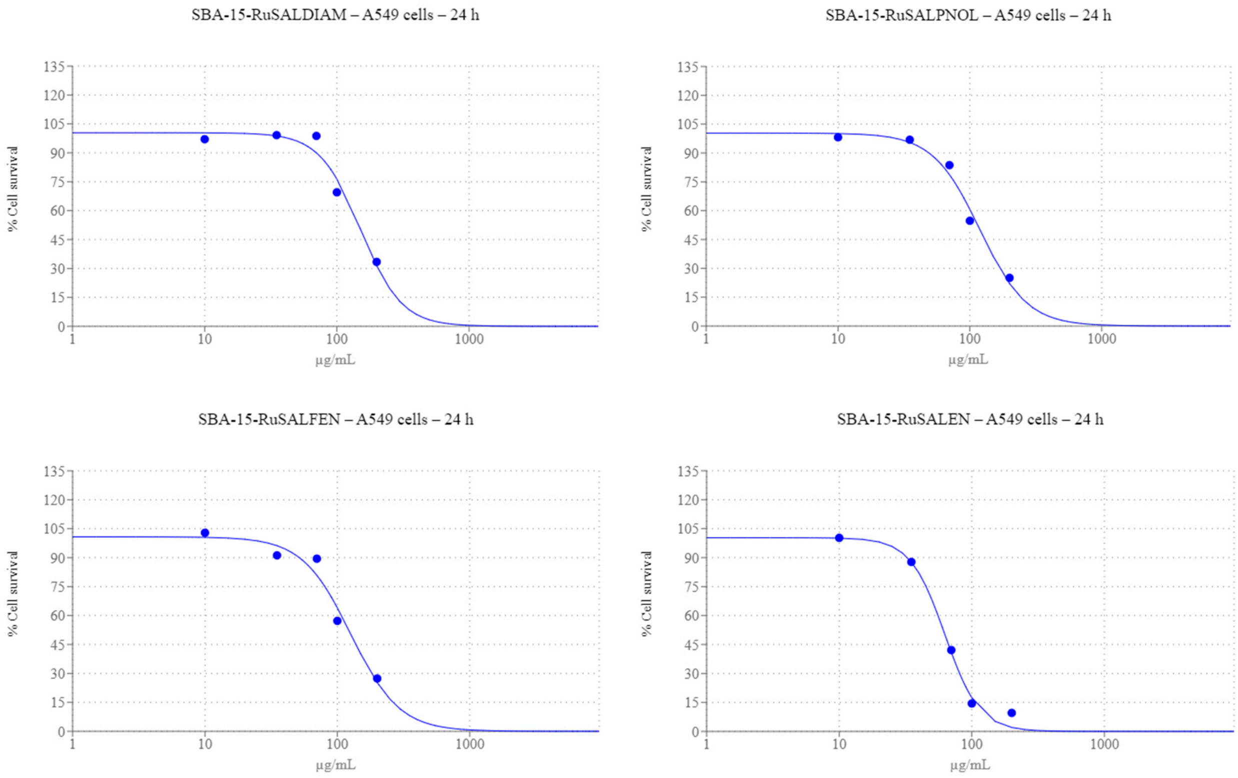

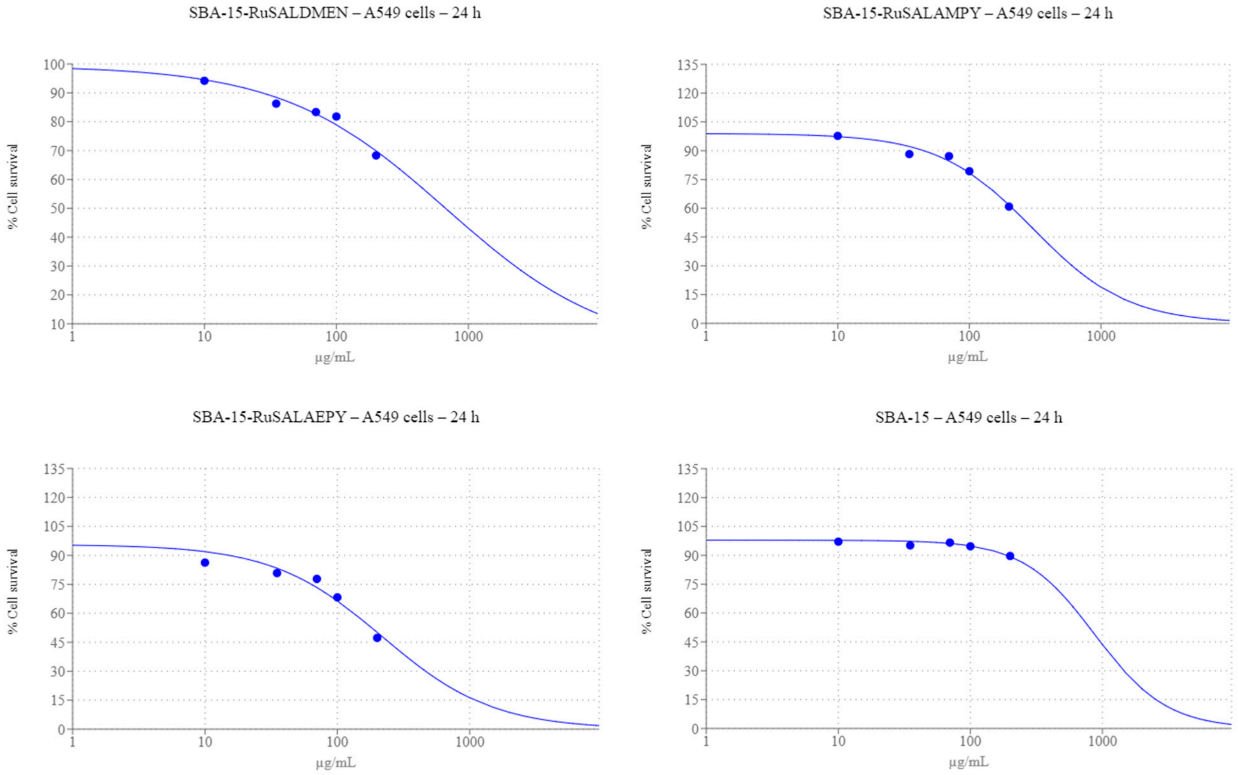

Figure S3. Cell survival graphs (%) obtained by MTT assay for Ru-containing hybrid materials after 24 h of incubation with A549 lung tumor cells.

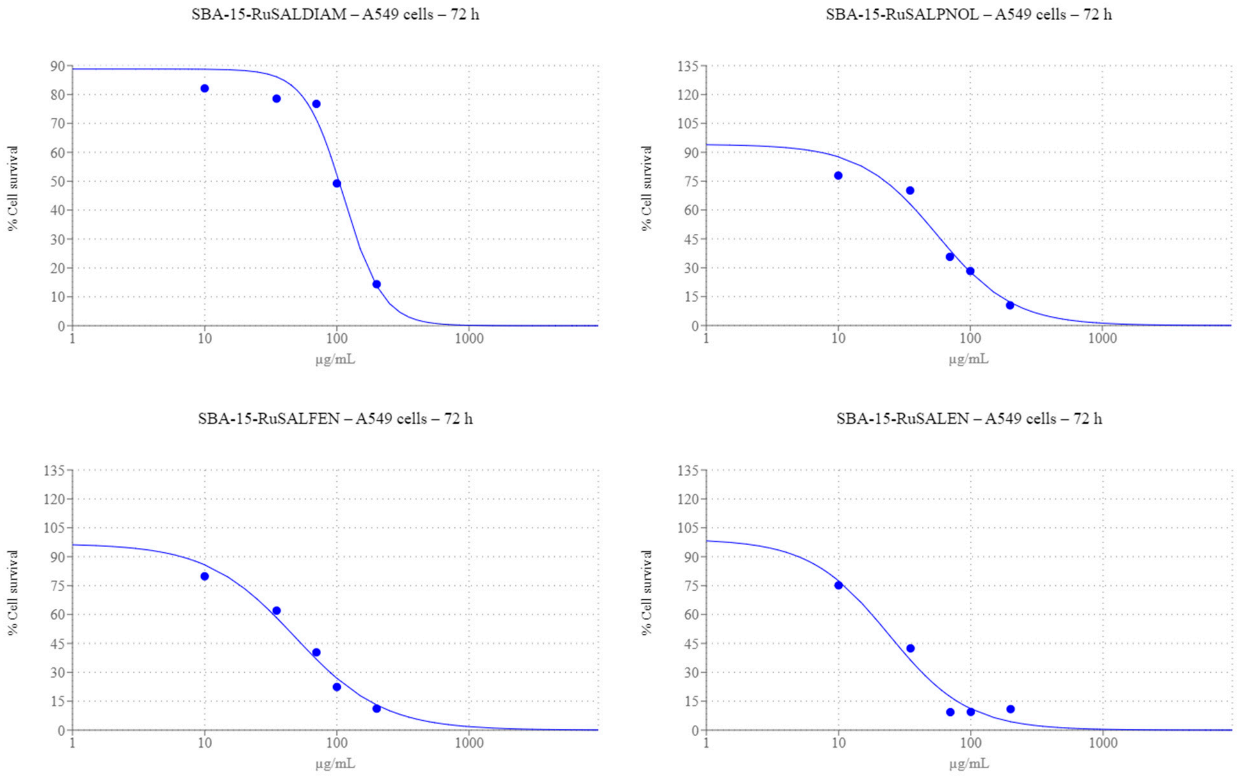

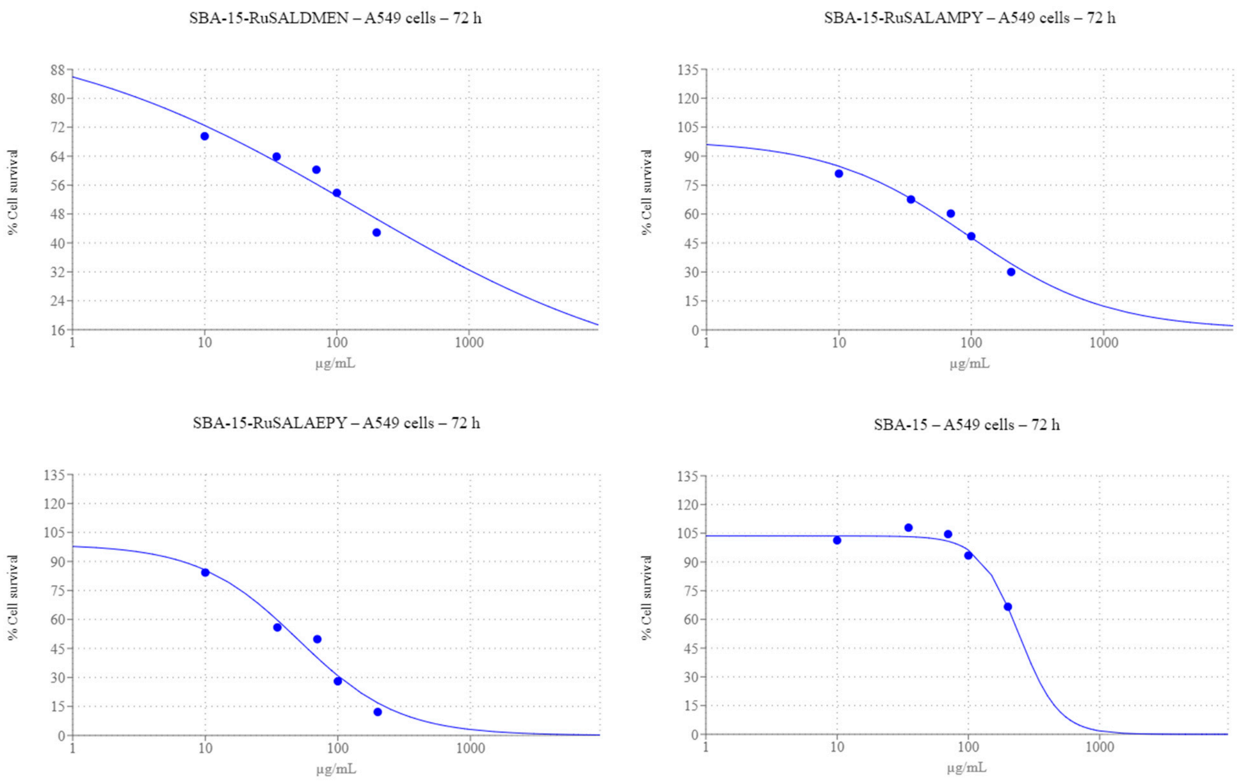

Figure S4. Cell survival graphs (%) obtained by MTT assay for Ru-containing hybrid materials after 72 h of incubation with A549 lung tumor cells.

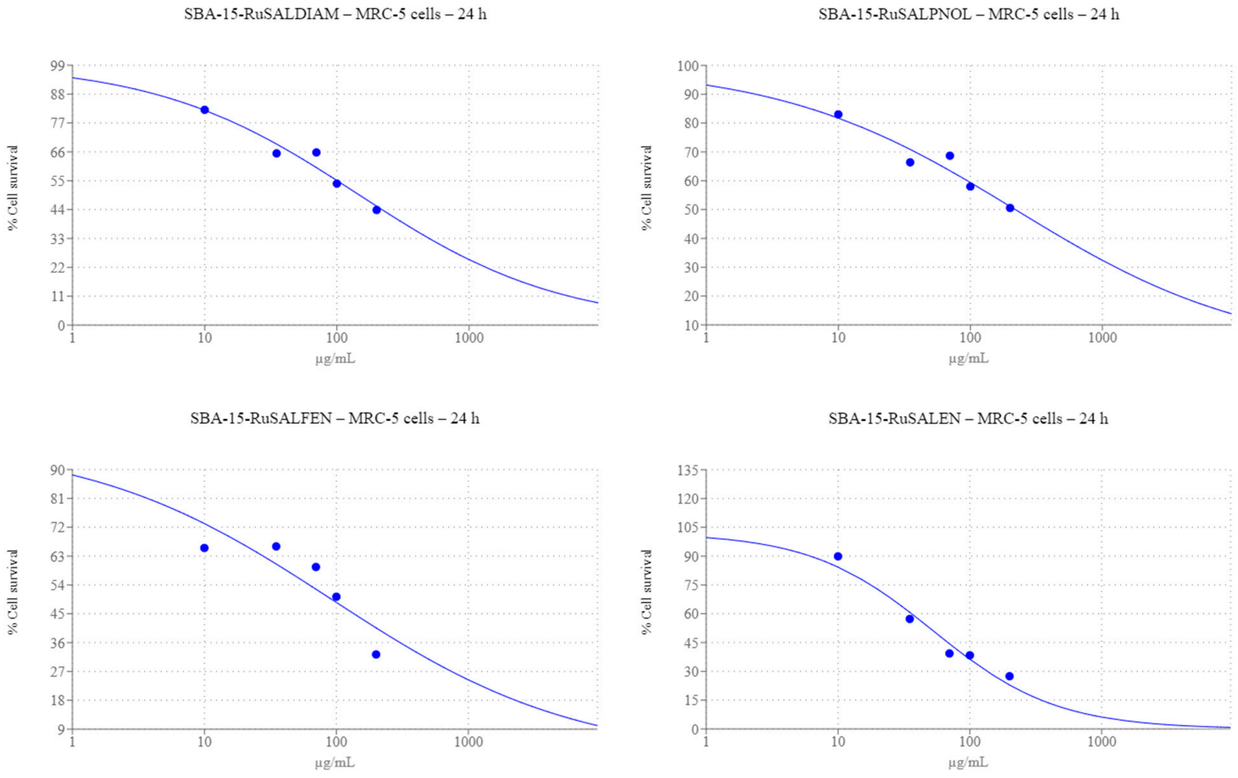

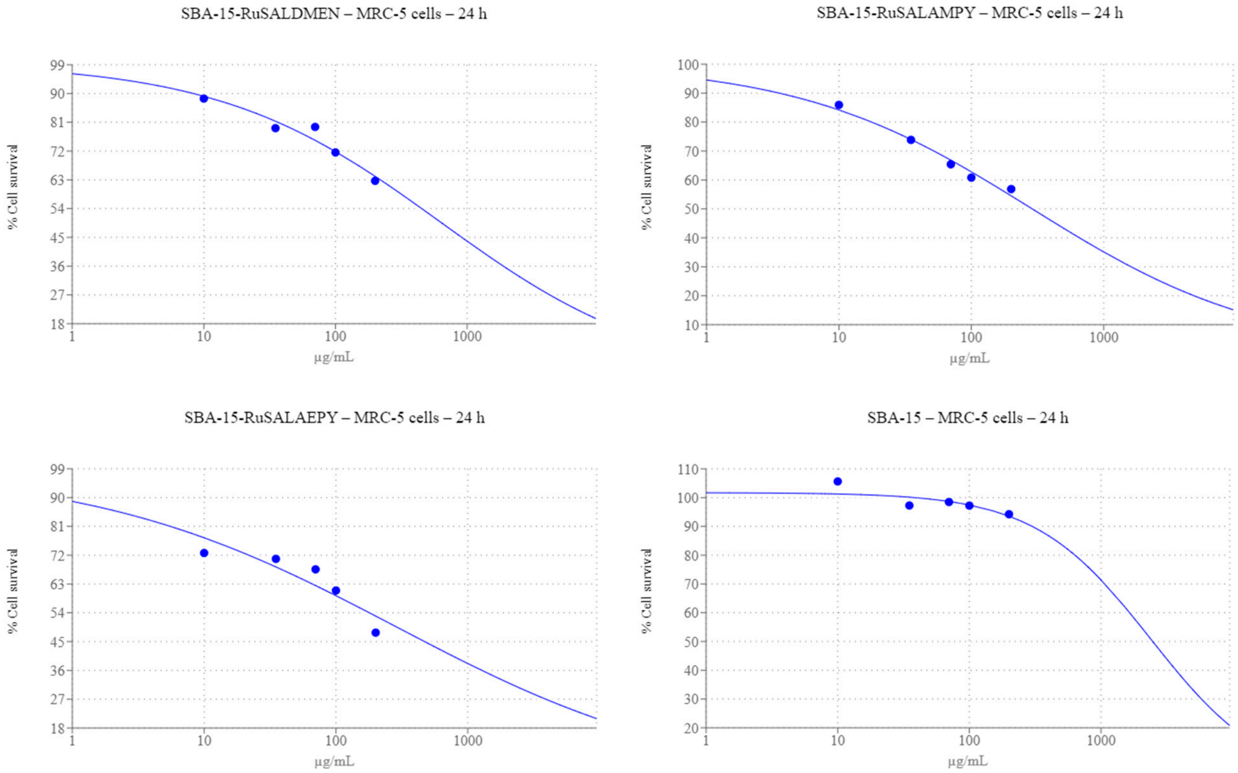

Figure S5. Cell survival graphs (%) obtained by MTT assay for Ru-containing hybrid materials after 24 h of incubation with MRC-5 lung non-tumoral cells.

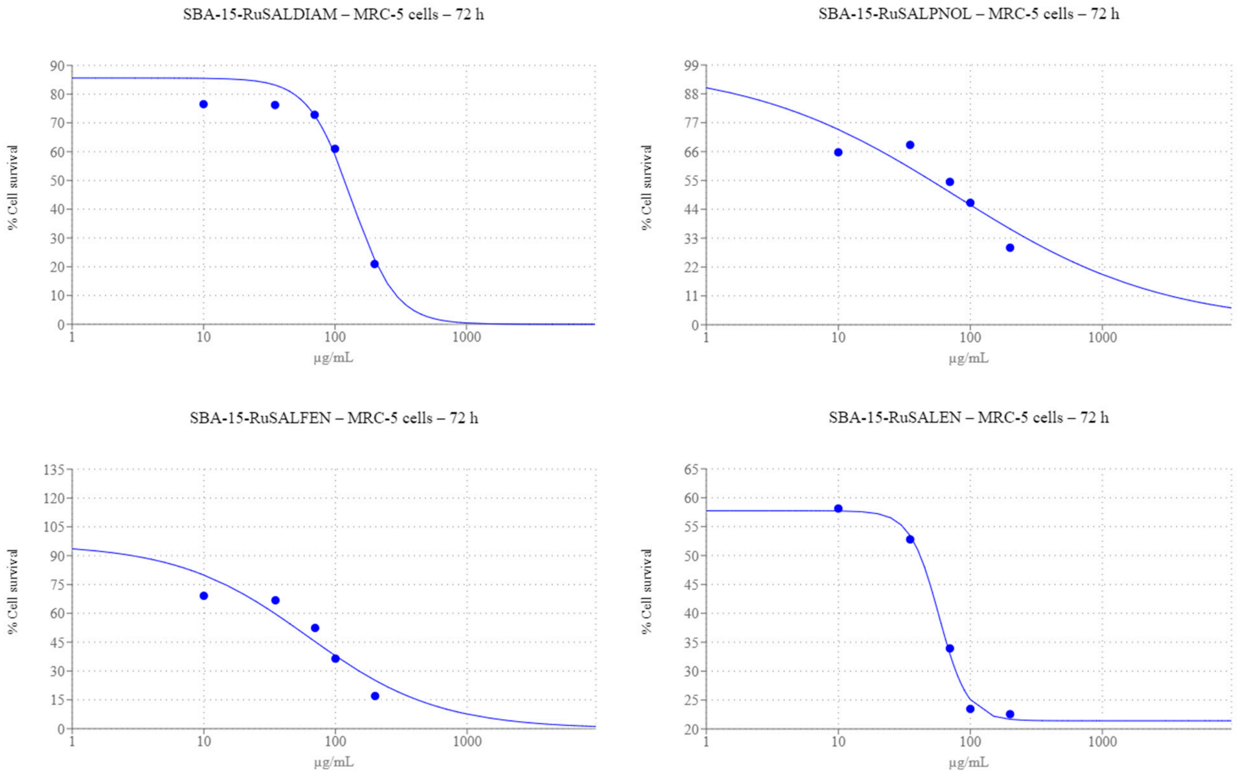

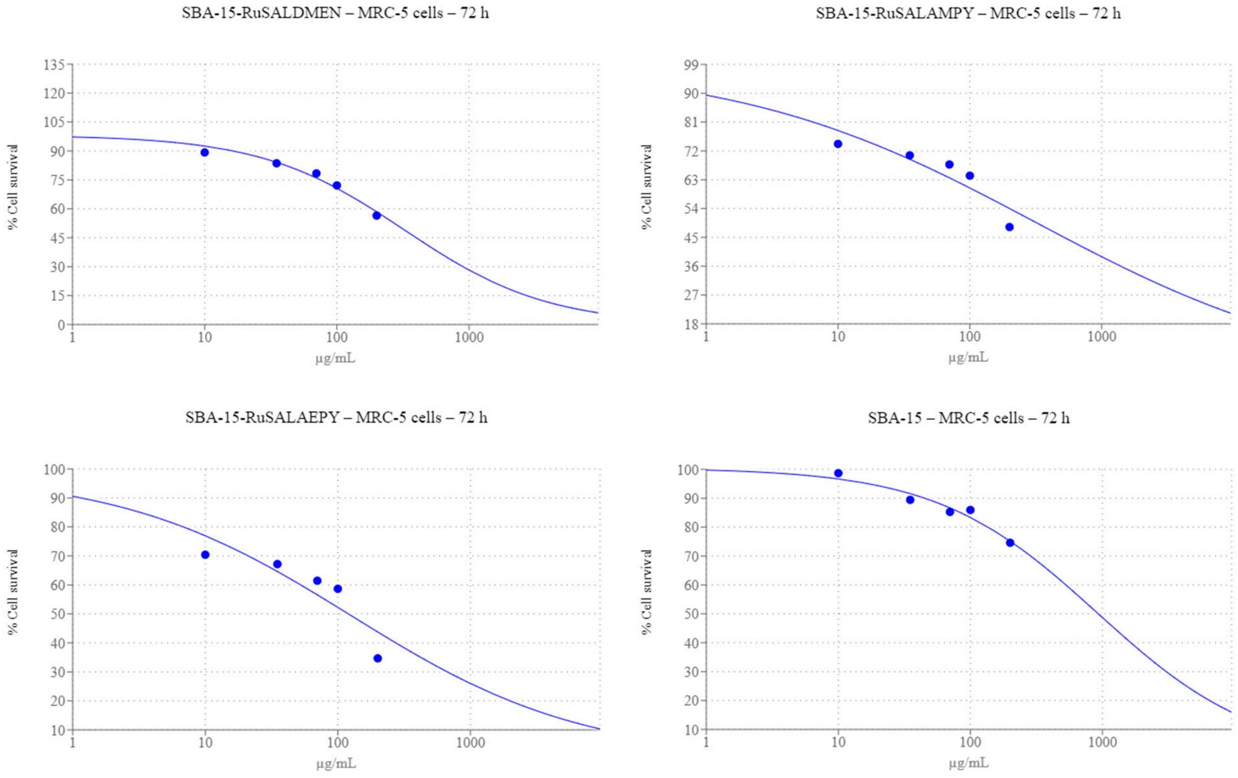

Figure S6. Cell survival graphs (%) obtained by MTT assay for Ru-containing hybrid materials after 72 h of incubation with MRC-5 lung non-tumoral cells.
